# Supplementary material for: Insulin and epidermal signals independently shape sexually dimorphic neurite branching in C. elegans
Source: EMBO Rep. 2025 Oct 31;26(23):5859–76. doi: 10.1038/s44319-025-00608-0 (PMC12678580; doi:10.1038/s44319-025-00608-0)
Supplement: Supplementary file 1 — Appendix [file 44319_2025_608_MOESM1_ESM.pdf]

Appendix

Insulin and Epidermal Signals Independently Shape Sexually Dimorphic Neurite Branching in *C. elegans*

Jia-Bin Yang[1], Rui-Tsung Chen[1], Yun-Yu Chen[1], Yun-Hsien Lin[1], Chun-Hao Chen[1,2,*]

[1] Institute of Molecular and Cellular Biology, College of Life Science, National Taiwan University. No. 1, Sec. 4, Roosevelt Rd., Taipei 10617, Taiwan.

[2] Department of Life Science, College of Life Science, National Taiwan University. No. 1, Sec. 4, Roosevelt Rd., Taipei 10617, Taiwan.

* Correspondence: chunhaochen@ntu.edu.tw

Table of Content

APPENDIX FIGURE S1. MOLECULAR CHARACTERIZATION OF PVP BRANCHES. 2

APPENDIX FIGURE S2. F-ACTIN DISTRIBUTION IN PVP NEURONS. 3

APPENDIX FIGURE S3. CELL-SPECIFIC VULVAL ABLATION BY THE MITO-MINISOG SYSTEM. 4

APPENDIX FIGURE S4. TRA-1 EXPRESSION IN PVP NEURONS AT VARIOUS DEVELOPMENTAL STAGES. 5

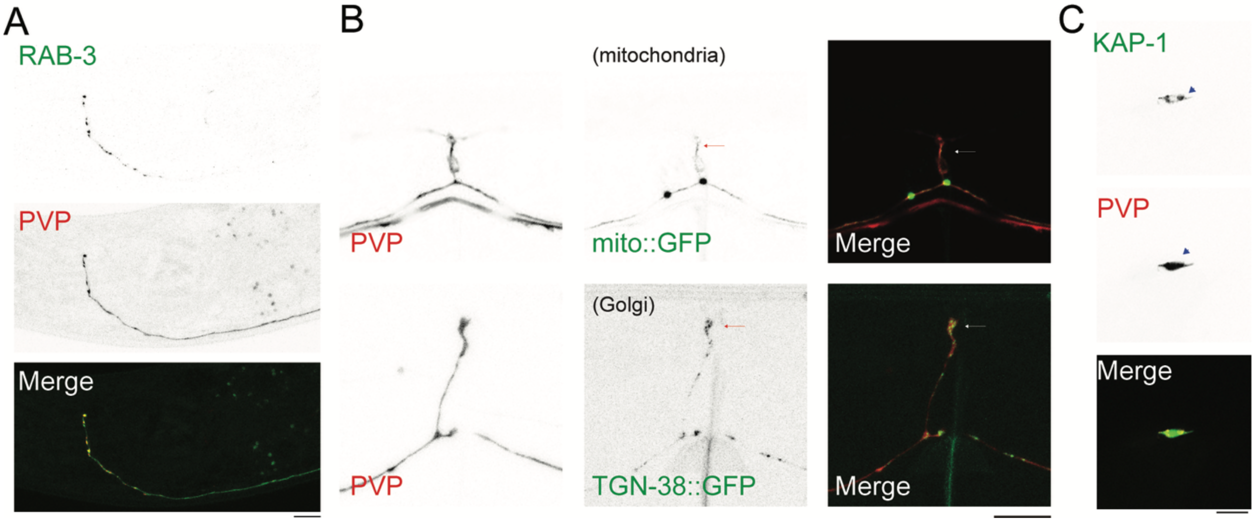

Appendix Figure S1. Molecular Characterization of PVP Branches.

(A-C) Z-projection of confocal fluorescent images or epifluorescent images of PVP neurons and markers for mitochondria (mito::GFP) and trans-Golgi (TGN-38::GFP) in the *chcEx072[Pocr-3::mito::GFP] and chcEx073[Pocr-3::TGN-38::GFP]* transgenic hermaphrodites. Arrowheads indicate cell soma. Arrows indicate PVP branches. Scale bar=10 µm.

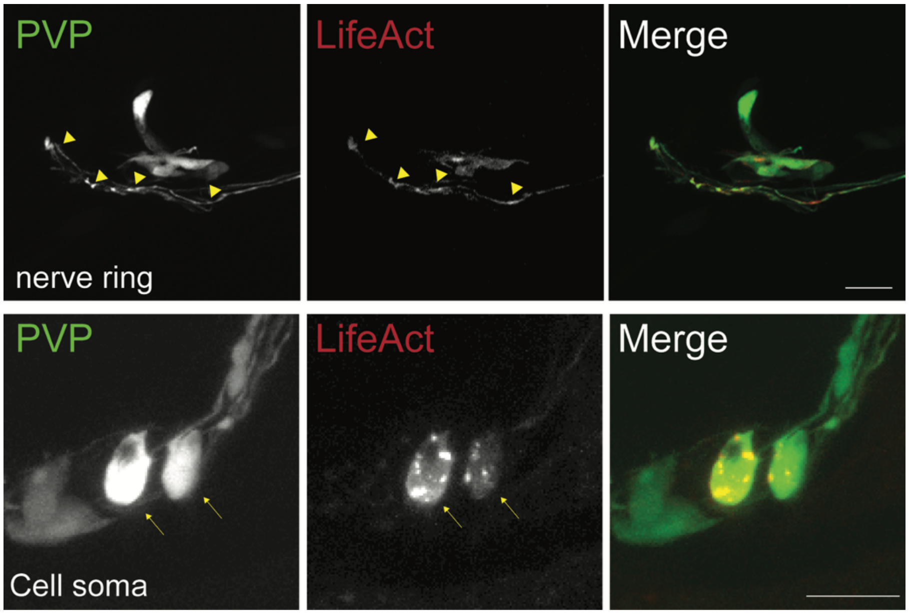

Appendix Figure S2. F-actin Distribution in PVP Neurons.

Z-projection of confocal fluorescent images of LifeAct in PVP processes and cell soma in the *chcEx070[Pocr-3::LifeAct::mKate]*. Arrowheads label nerve ring. Arrows label the cell soma. Scale bar=10 μm.

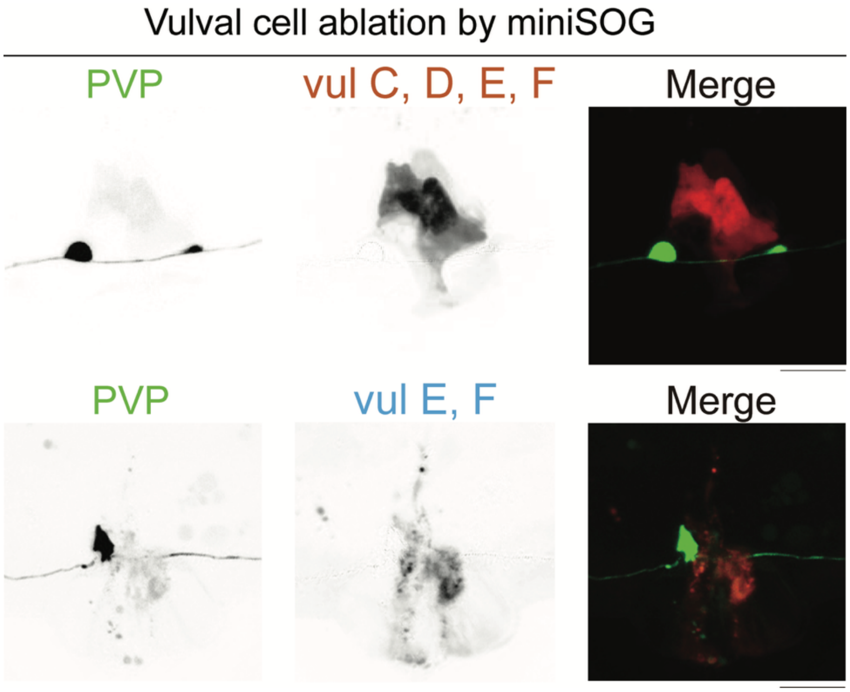

Appendix Figure S3. Cell-specific Vulval Ablation by the Mito-miniSOG system.

Z-projection of confocal fluorescent images in the *chcSi1* hermaphrodites with ablated vulval epithelium cells in the *chcEx162 (Pdaf-6::mito::miniSOG::SL2::mKate)* and *chcEx160 (Pcdh-3:: mito::miniSOG::SL2::mKate)*. Scale bar=10 μm.

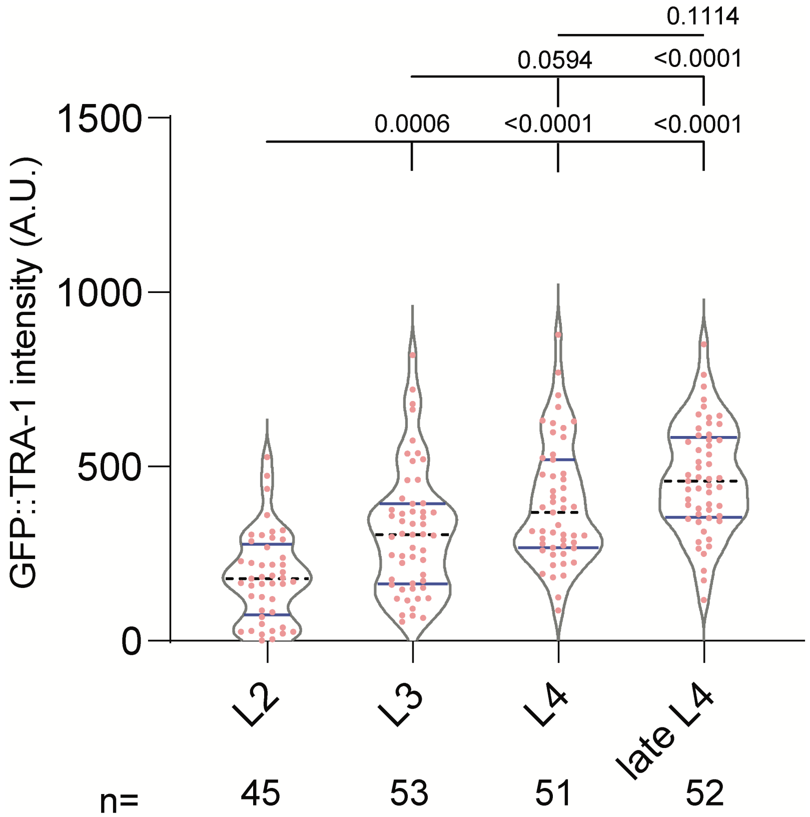

Appendix Figure S4. TRA-1 Expression in PVP Neurons at Various Developmental Stages.

TRA-1 signal labelled by *tra-1(ez72 [biotag::GFP::TEV::3xFlag::TRA-1])* in PVP cell soma at larva stages. One-way ANOVA with Tukey correction. N number and P value are indicated. N indicates the number of biological repeats in this figure.
