# Supplementary figures and images for: Insulin and epidermal signals independently shape sexually dimorphic neurite branching in C. elegans
Source: EMBO Rep. 2025 Oct 31;26(23):5859–76. doi: 10.1038/s44319-025-00608-0 (PMC12678580; doi:10.1038/s44319-025-00608-0)

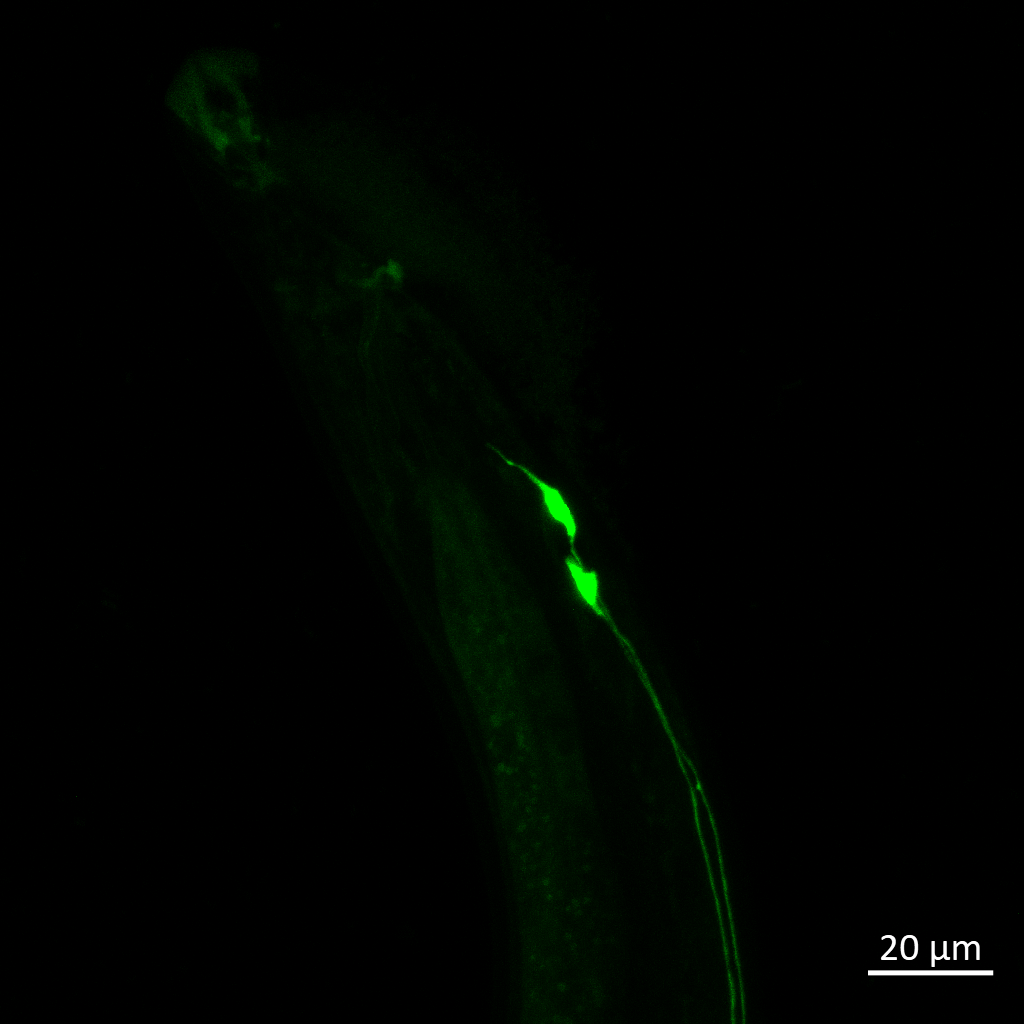

Supplement: Supplementary file 4 — Source data Fig. 1 [file 44319_2025_608_MOESM4_ESM.zip › Figure 1/1A/male 1.tif]

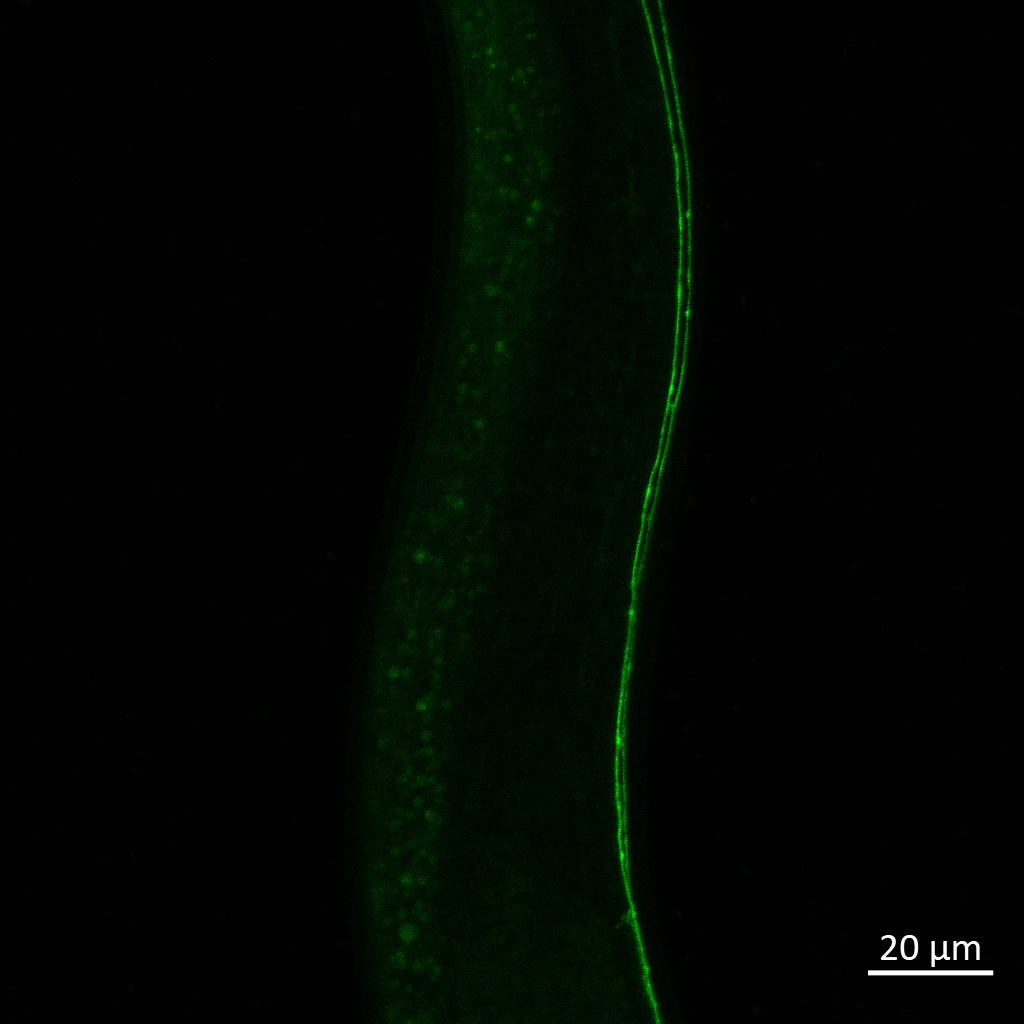

Supplement: Supplementary file 4 — Source data Fig. 1 [file 44319_2025_608_MOESM4_ESM.zip › Figure 1/1A/male 2.tif]

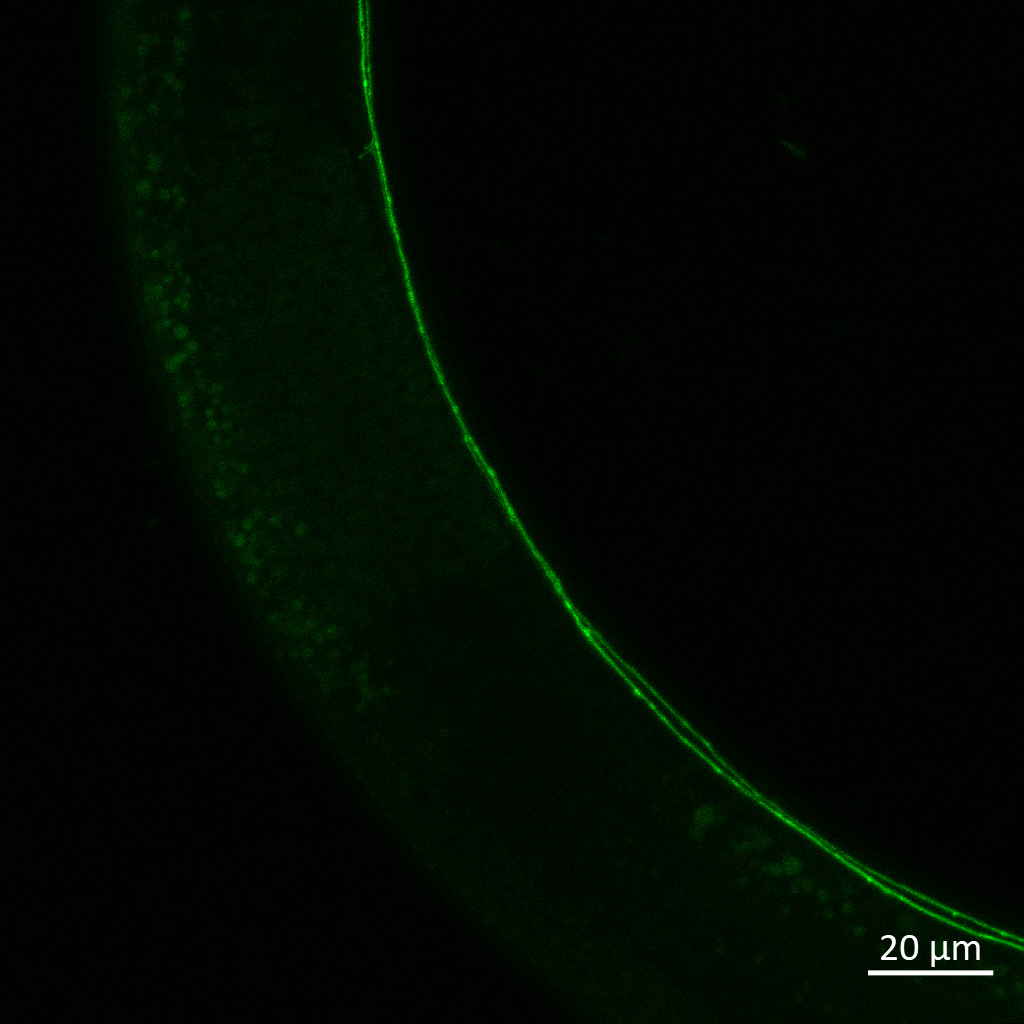

Supplement: Supplementary file 4 — Source data Fig. 1 [file 44319_2025_608_MOESM4_ESM.zip › Figure 1/1A/male 3.tif]

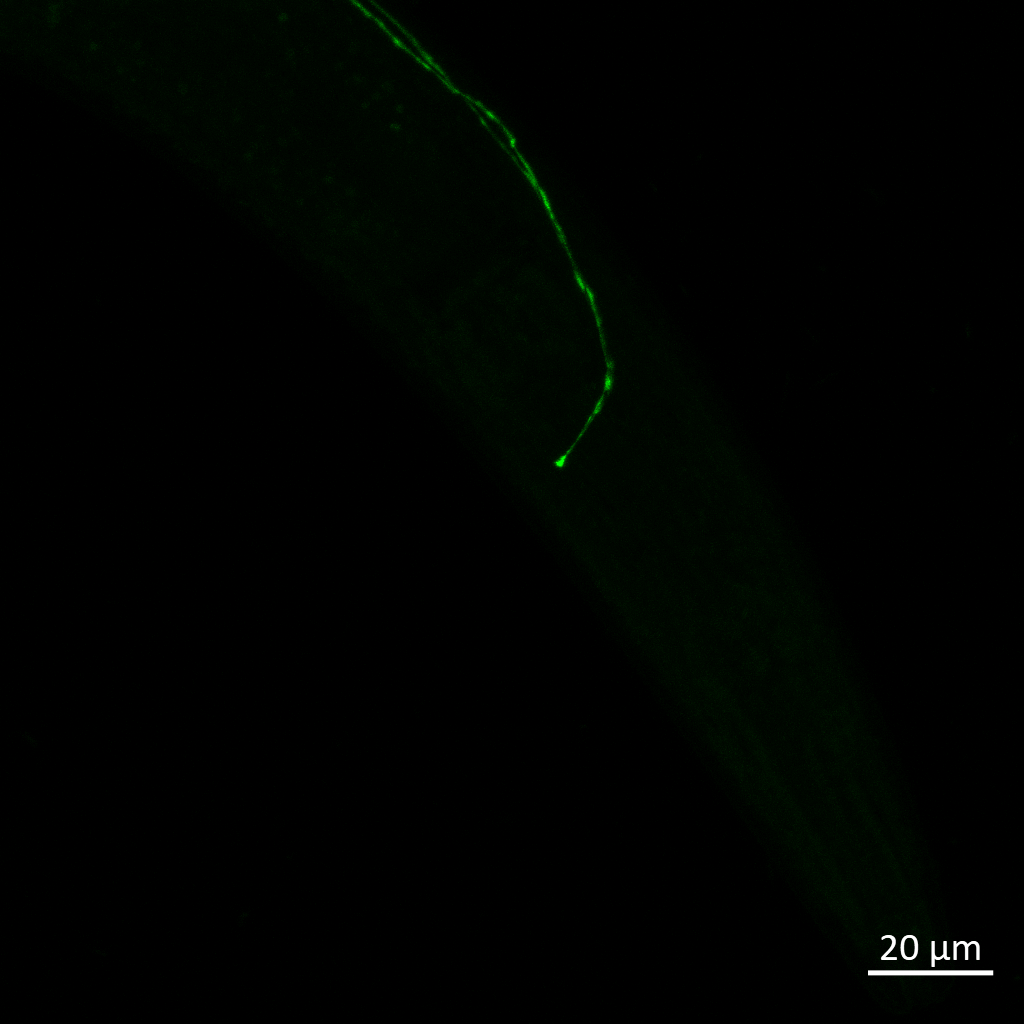

Supplement: Supplementary file 4 — Source data Fig. 1 [file 44319_2025_608_MOESM4_ESM.zip › Figure 1/1A/male 7.tif]

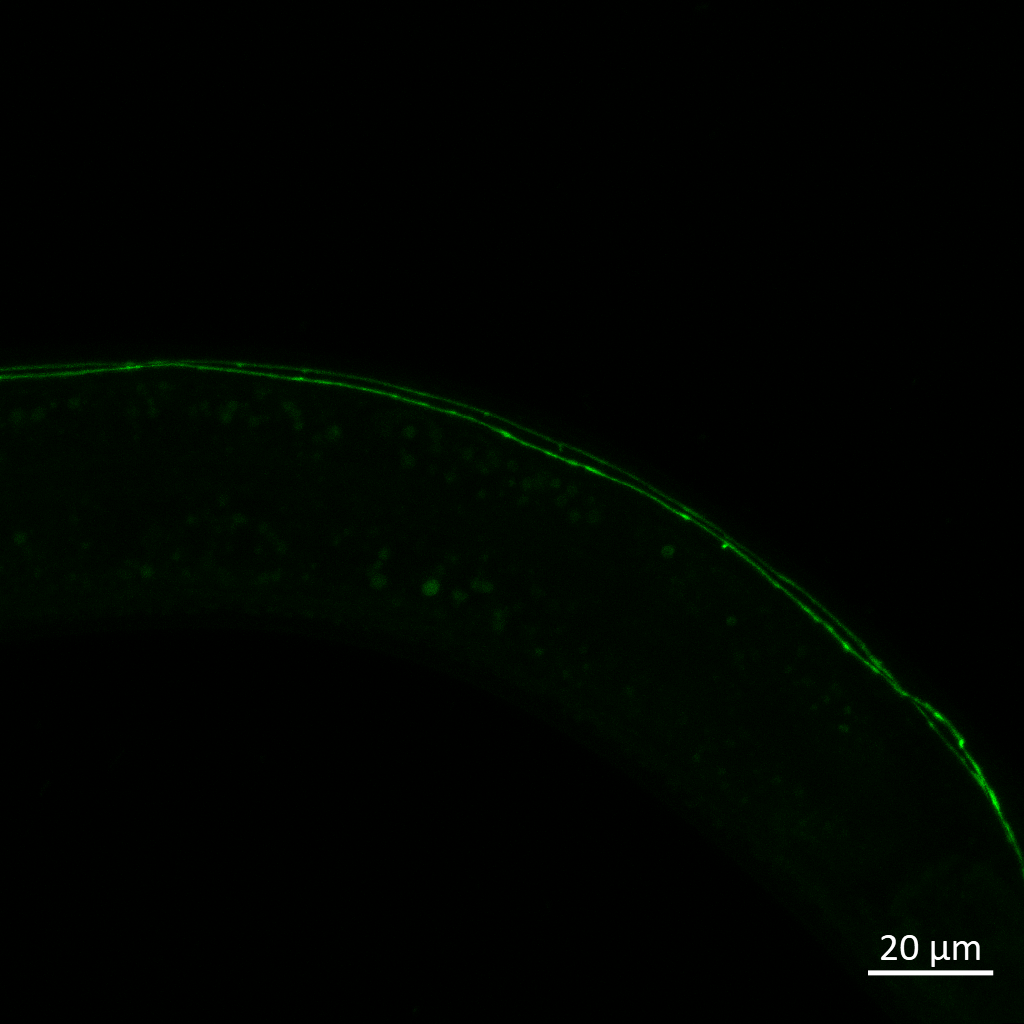

Supplement: Supplementary file 4 — Source data Fig. 1 [file 44319_2025_608_MOESM4_ESM.zip › Figure 1/1A/male 6.tif]

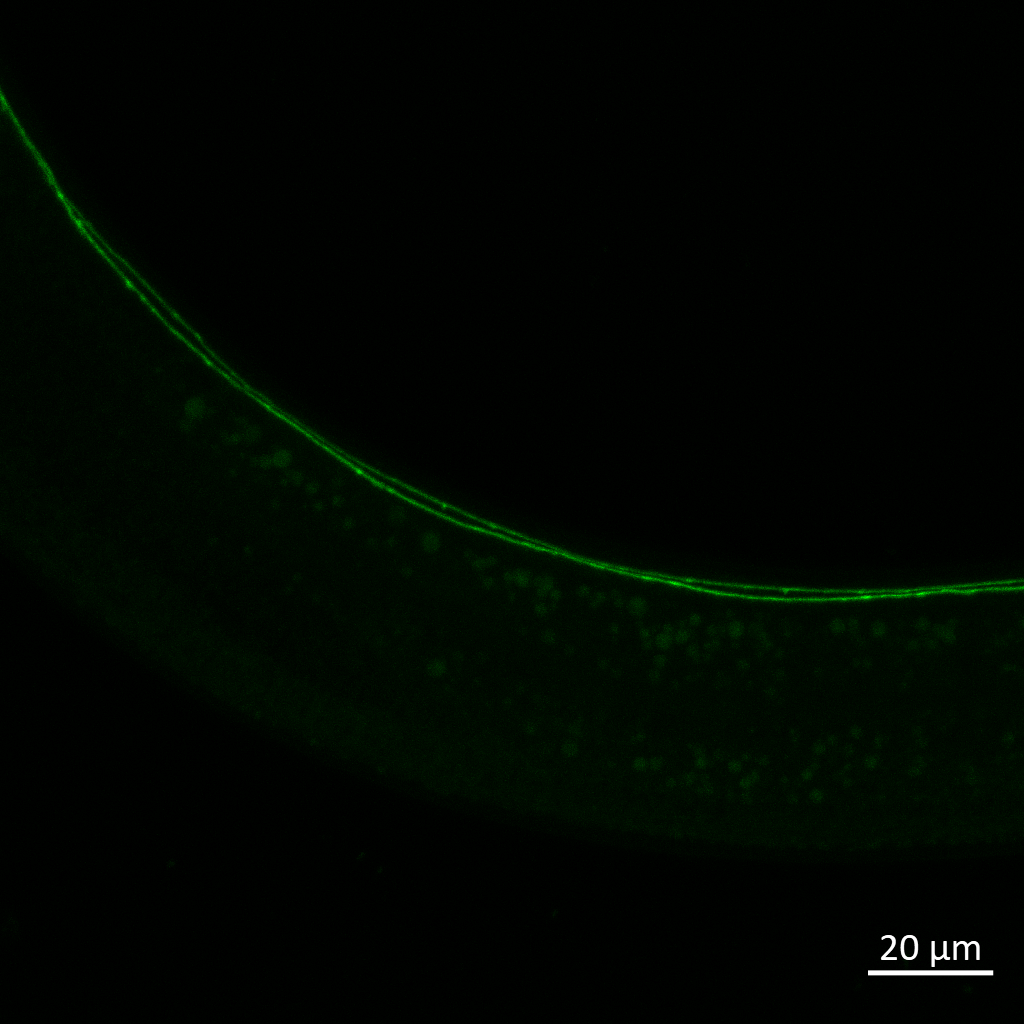

Supplement: Supplementary file 4 — Source data Fig. 1 [file 44319_2025_608_MOESM4_ESM.zip › Figure 1/1A/male 4.tif]

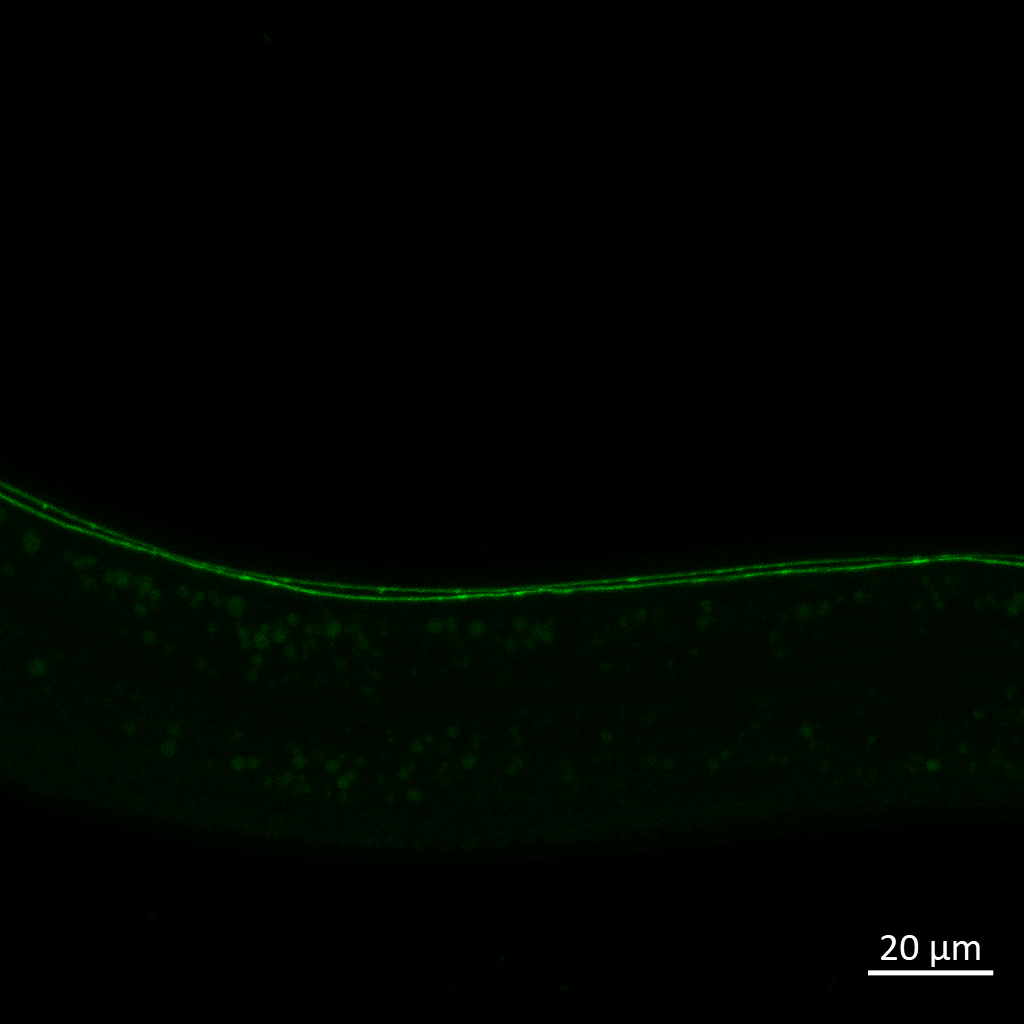

Supplement: Supplementary file 4 — Source data Fig. 1 [file 44319_2025_608_MOESM4_ESM.zip › Figure 1/1A/male 5.tif]

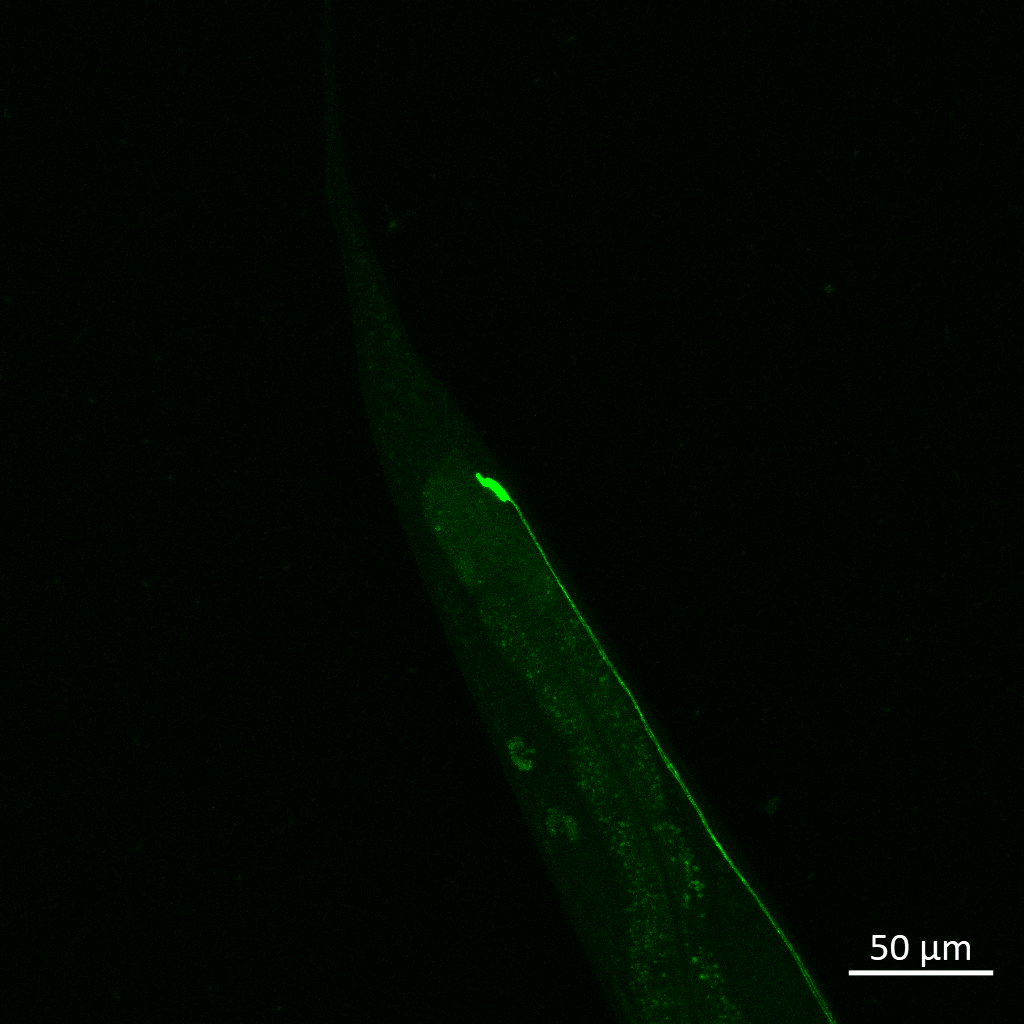

Supplement: Supplementary file 4 — Source data Fig. 1 [file 44319_2025_608_MOESM4_ESM.zip › Figure 1/1A/herm 1.tif]

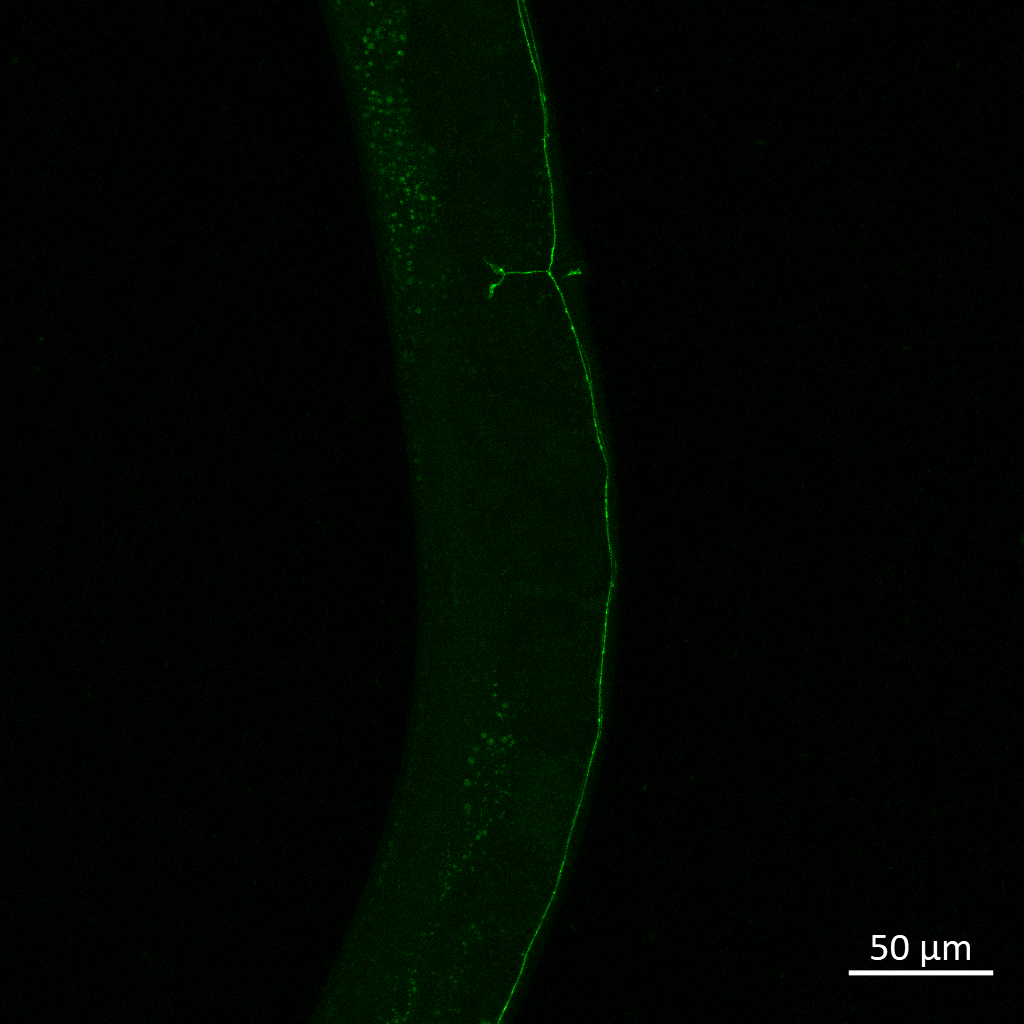

Supplement: Supplementary file 4 — Source data Fig. 1 [file 44319_2025_608_MOESM4_ESM.zip › Figure 1/1A/herm 3.tif]

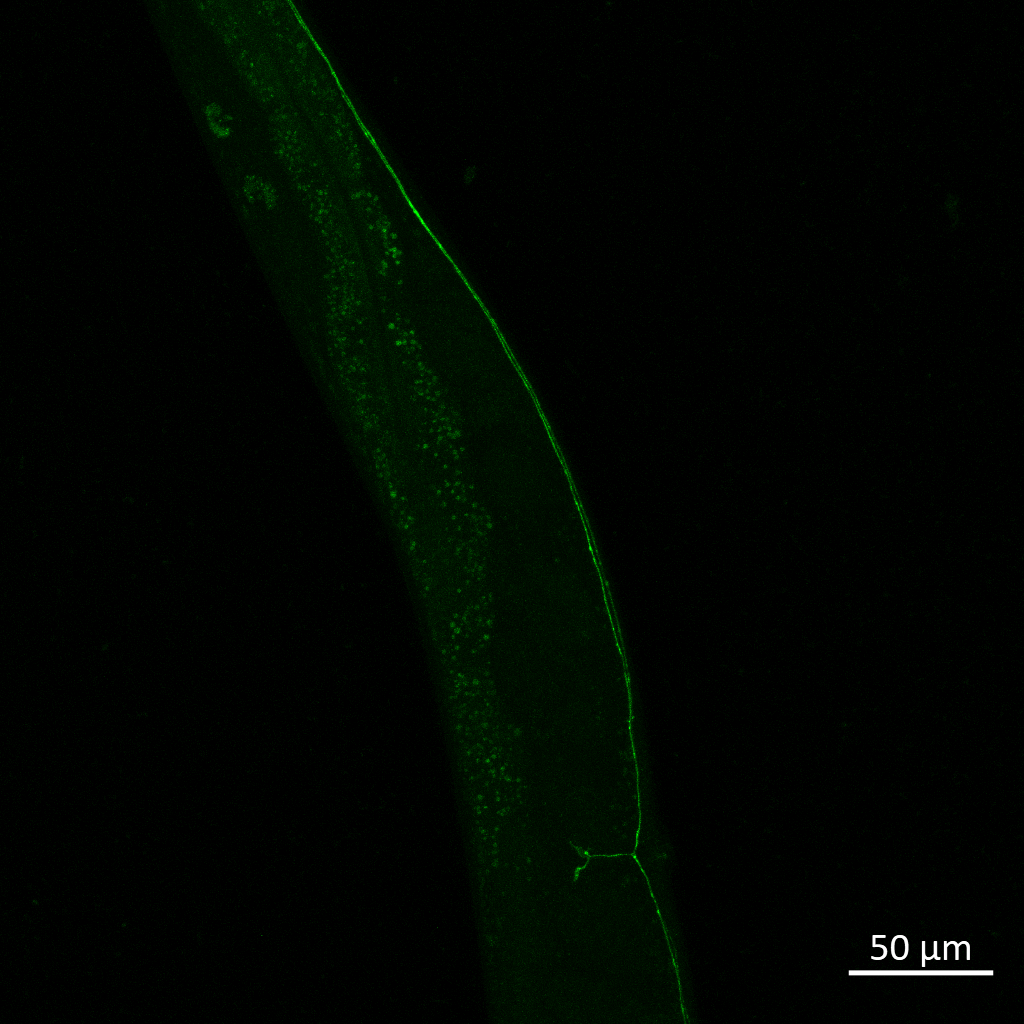

Supplement: Supplementary file 4 — Source data Fig. 1 [file 44319_2025_608_MOESM4_ESM.zip › Figure 1/1A/herm 2.tif]

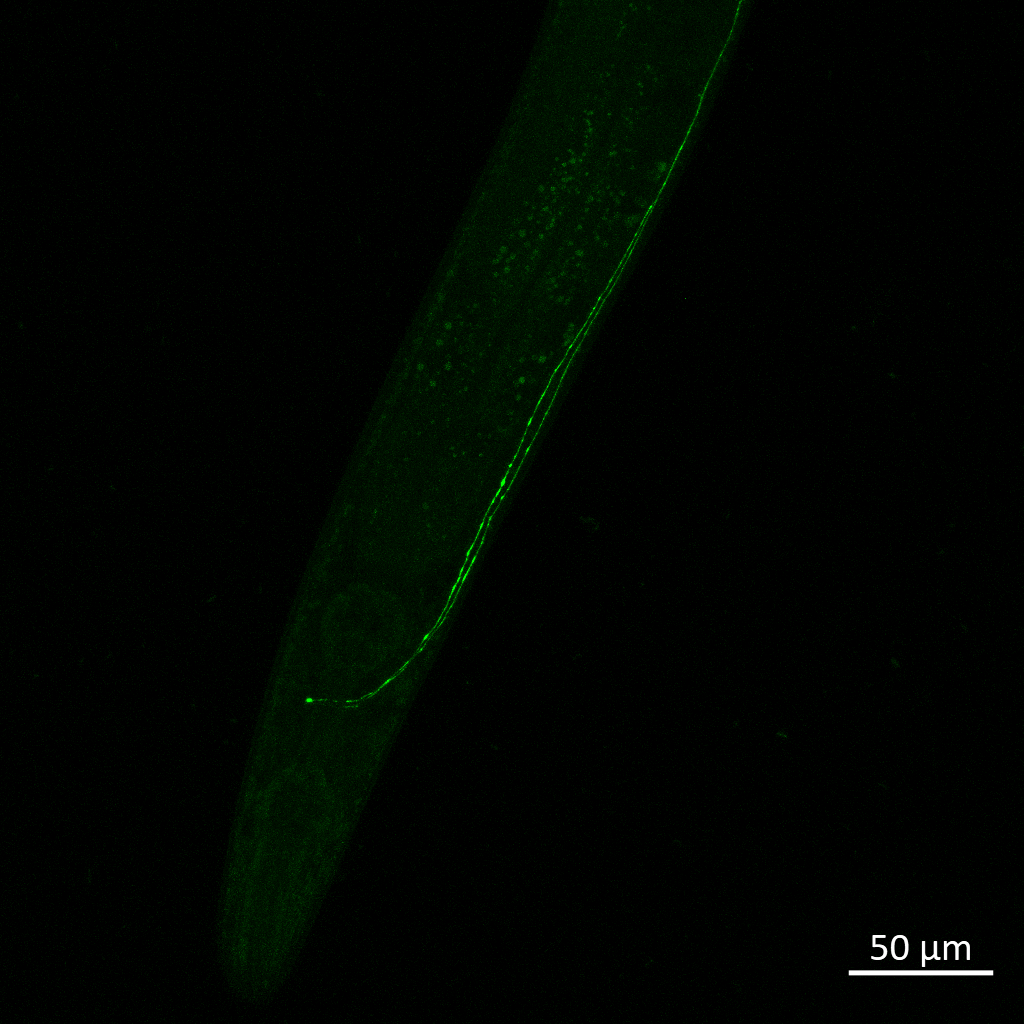

Supplement: Supplementary file 4 — Source data Fig. 1 [file 44319_2025_608_MOESM4_ESM.zip › Figure 1/1A/herm 4.tif]

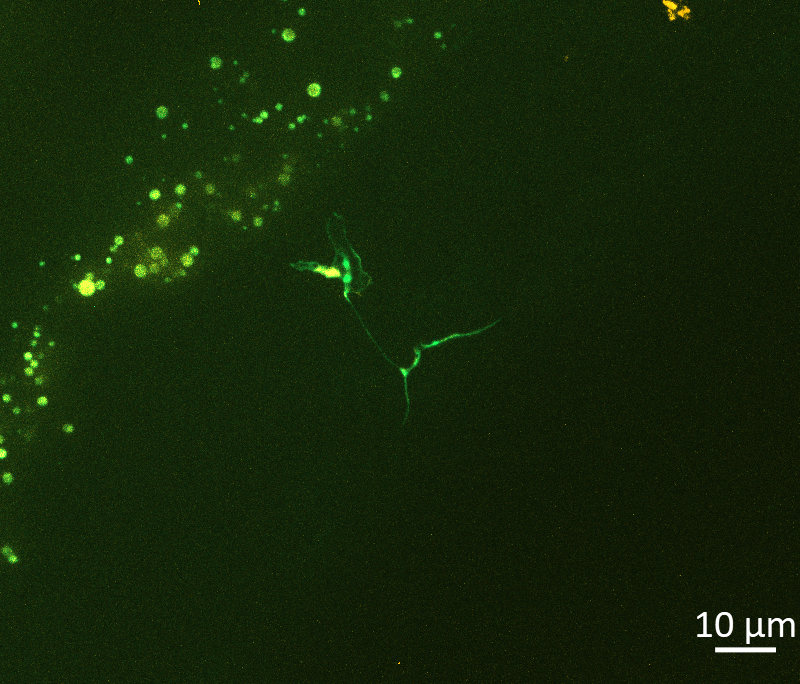

Supplement: Supplementary file 4 — Source data Fig. 1 [file 44319_2025_608_MOESM4_ESM.zip › Figure 1/1E/KAP-1.tif]

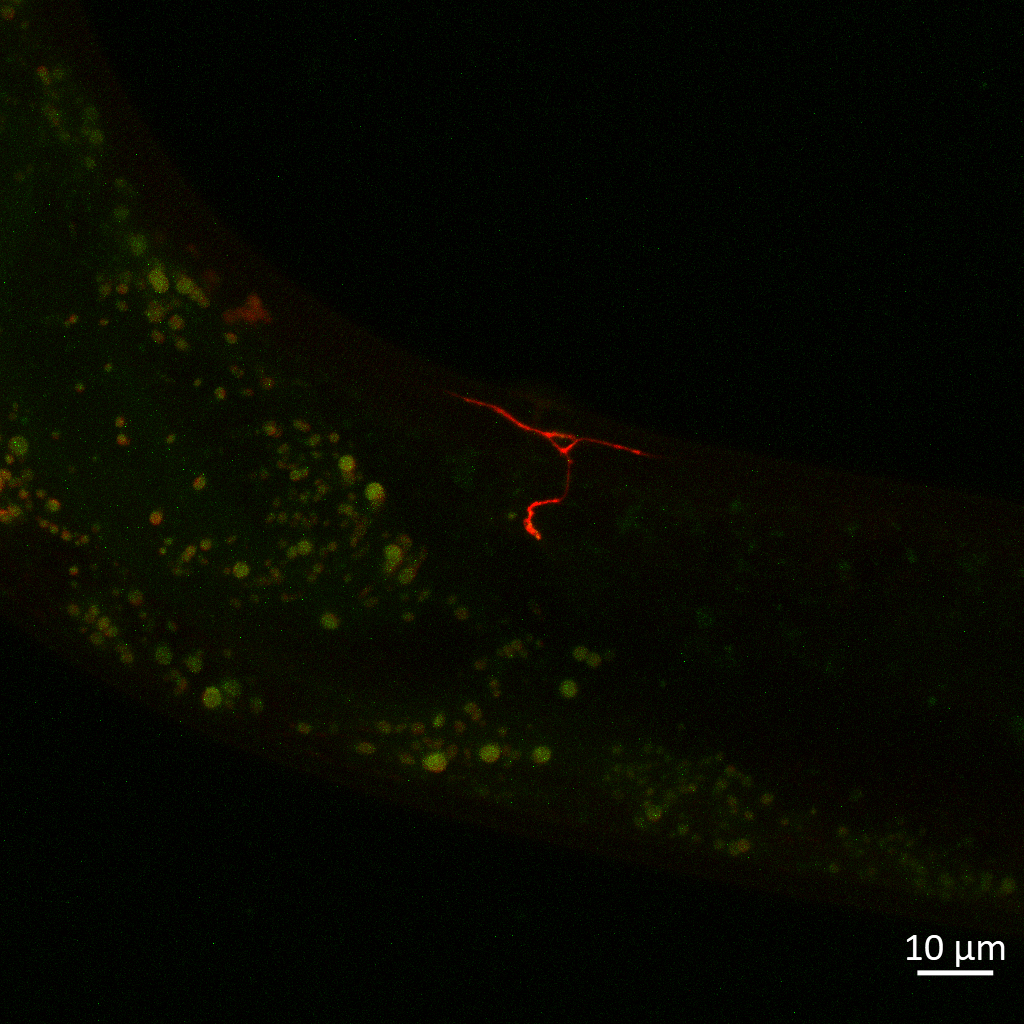

Supplement: Supplementary file 4 — Source data Fig. 1 [file 44319_2025_608_MOESM4_ESM.zip › Figure 1/1D/RAB-3.tif]

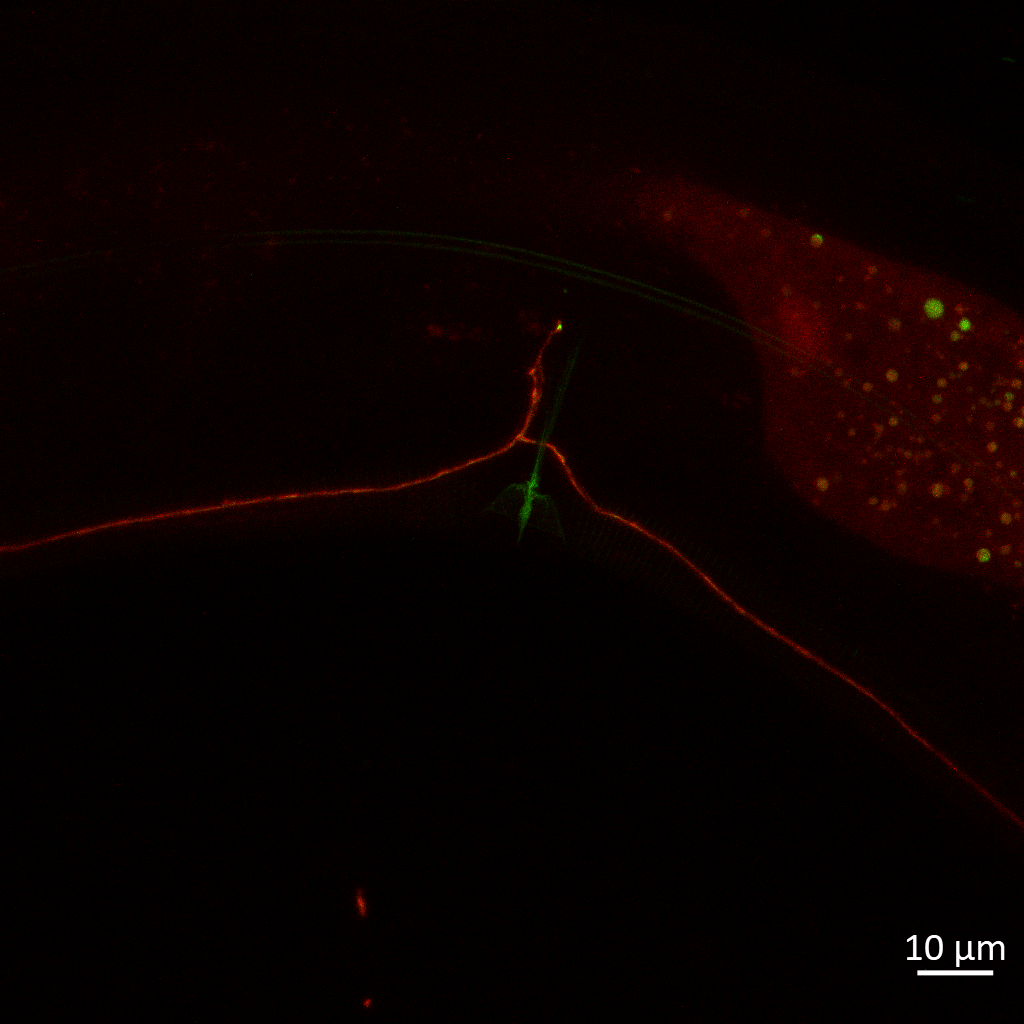

Supplement: Supplementary file 4 — Source data Fig. 1 [file 44319_2025_608_MOESM4_ESM.zip › Figure 1/1D/CLA-1.tif]

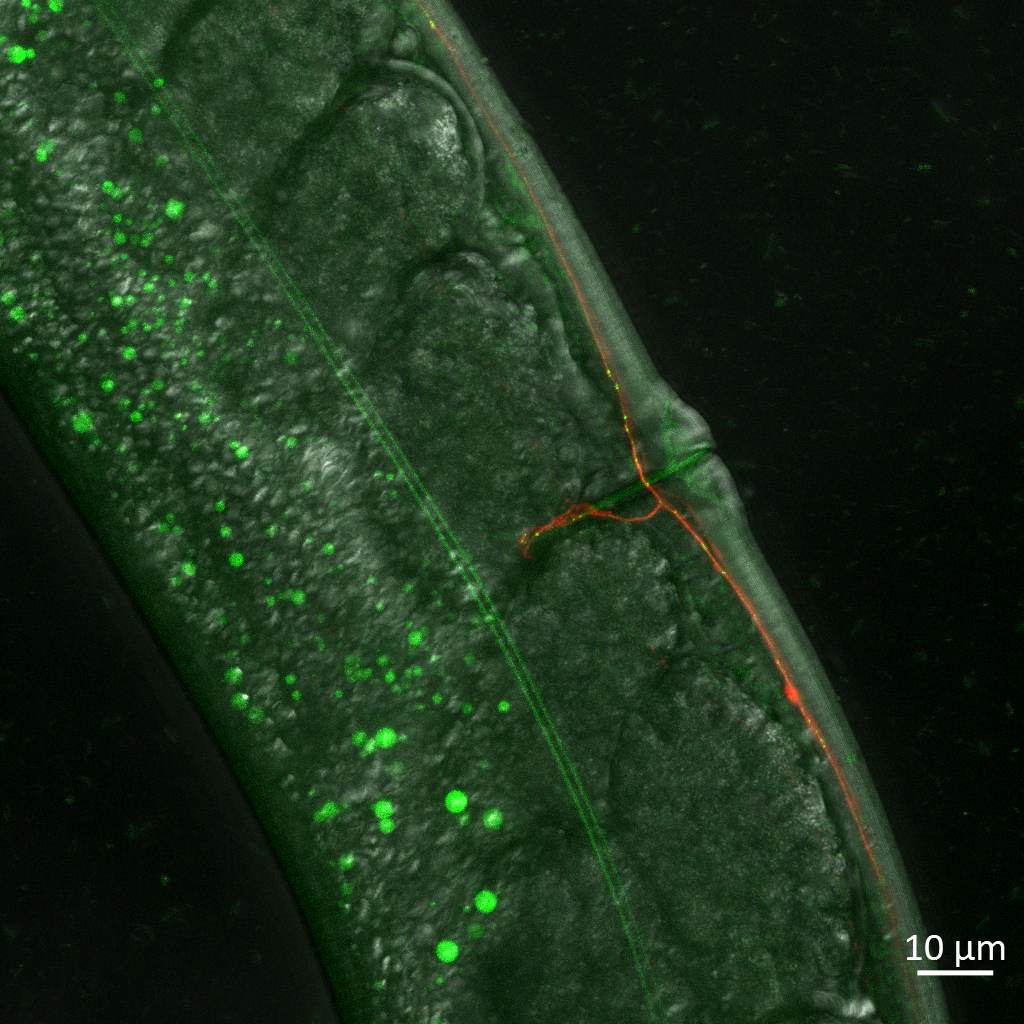

Supplement: Supplementary file 4 — Source data Fig. 1 [file 44319_2025_608_MOESM4_ESM.zip › Figure 1/1D/IDA-1.tif]

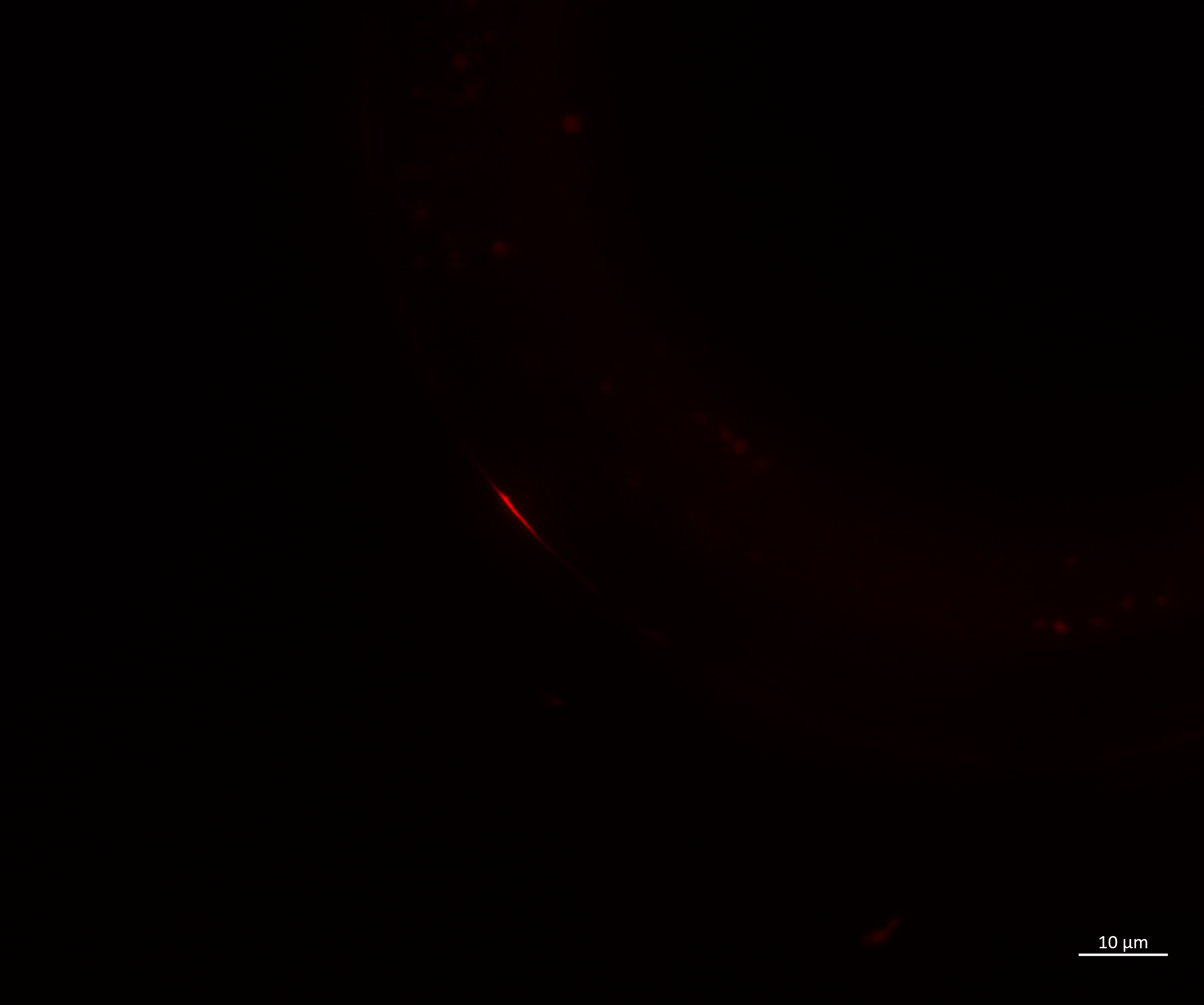

Supplement: Supplementary file 5 — Source data Fig. 2 [file 44319_2025_608_MOESM5_ESM.zip › Figure 2/2A/L4.tif]

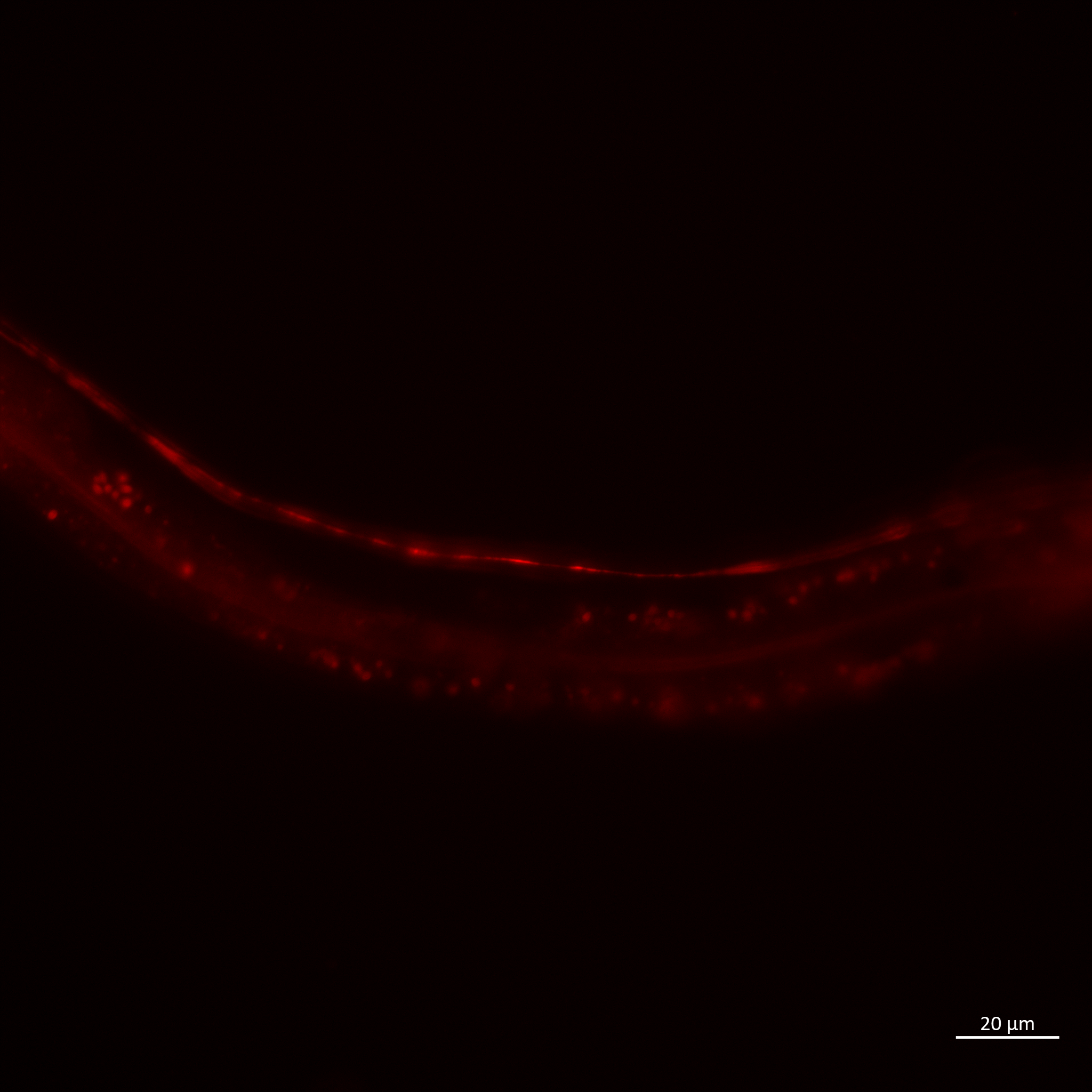

Supplement: Supplementary file 5 — Source data Fig. 2 [file 44319_2025_608_MOESM5_ESM.zip › Figure 2/2A/L3-1.tif]

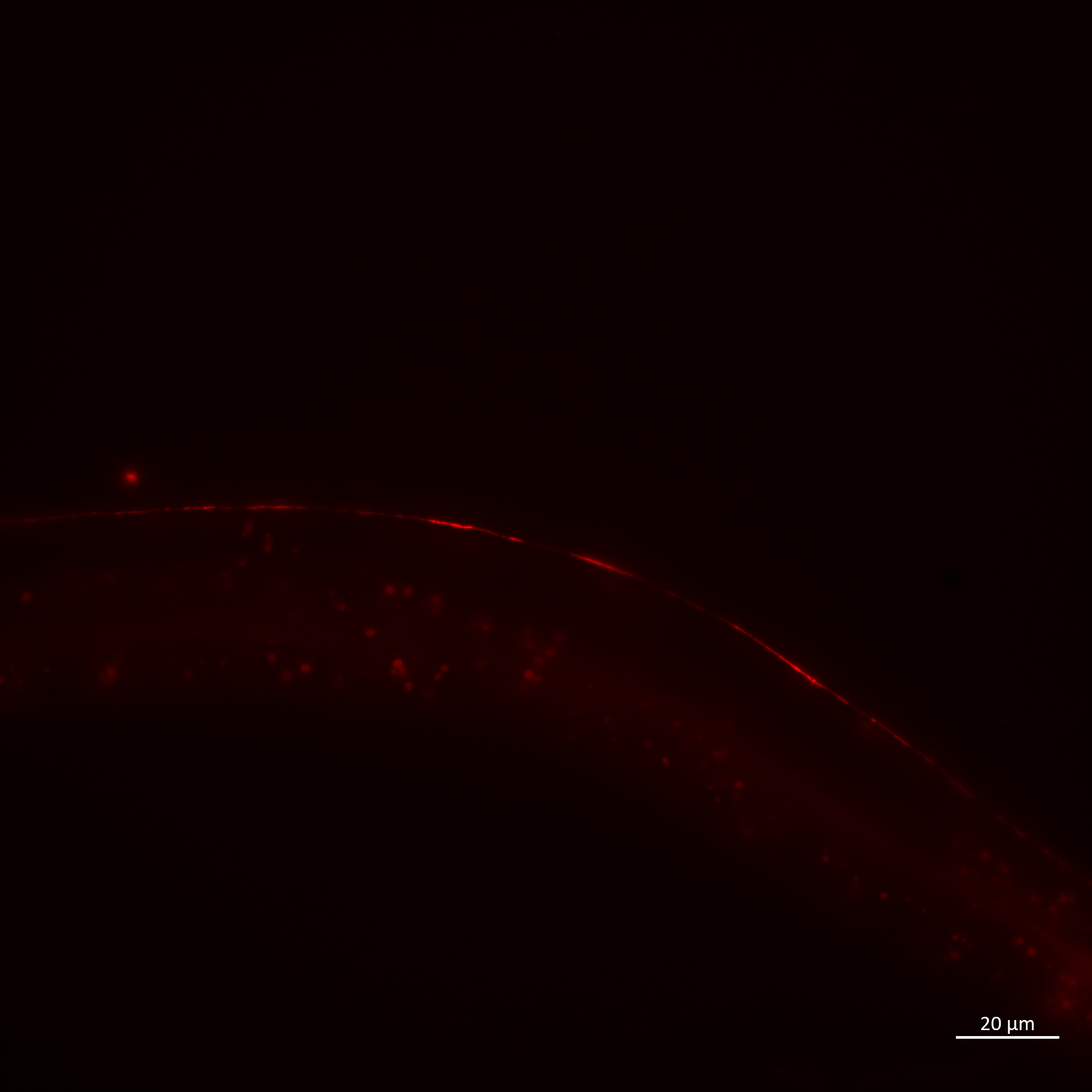

Supplement: Supplementary file 5 — Source data Fig. 2 [file 44319_2025_608_MOESM5_ESM.zip › Figure 2/2A/L3-2.tif]

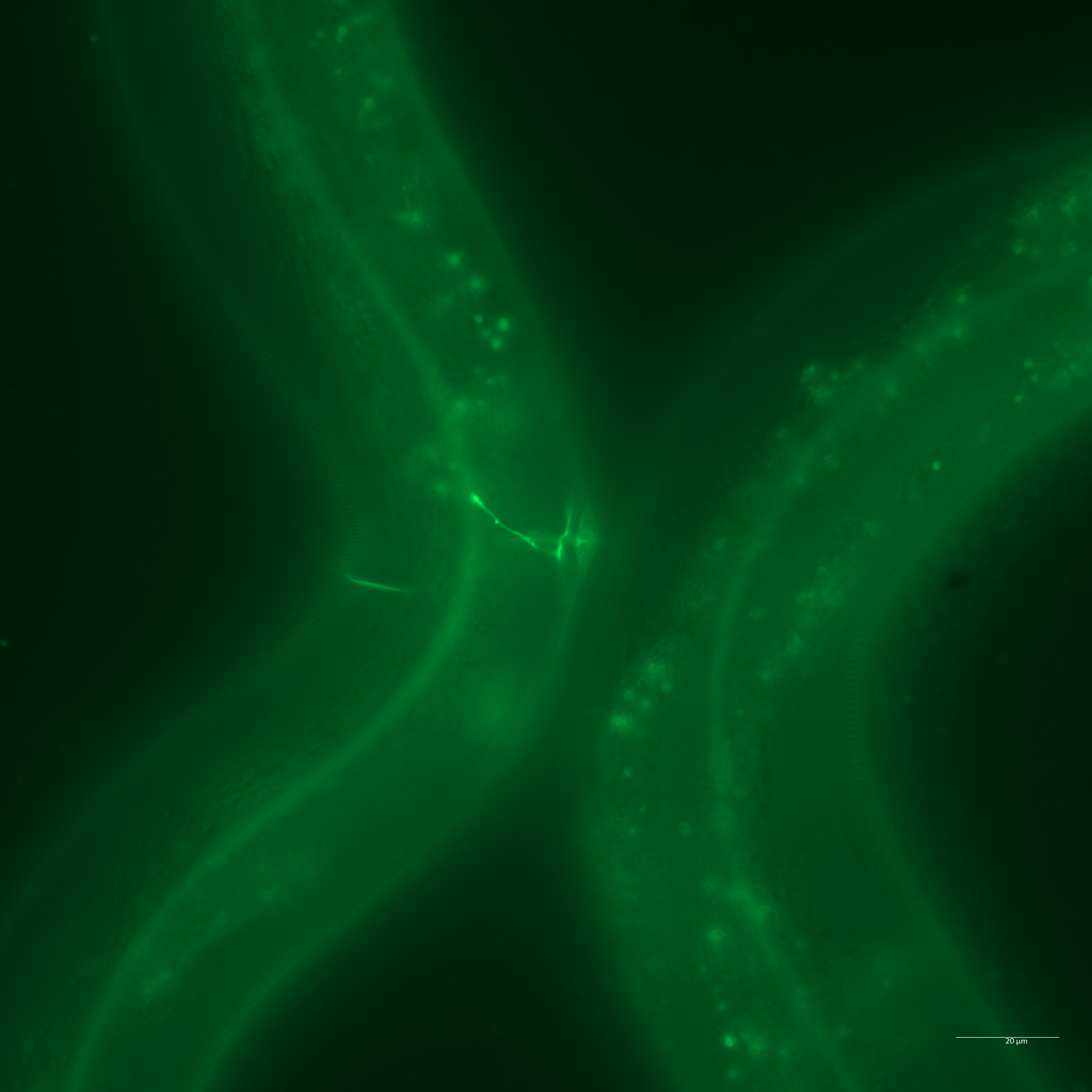

Supplement: Supplementary file 5 — Source data Fig. 2 [file 44319_2025_608_MOESM5_ESM.zip › Figure 2/2F/rod to wing - 0'.tif]

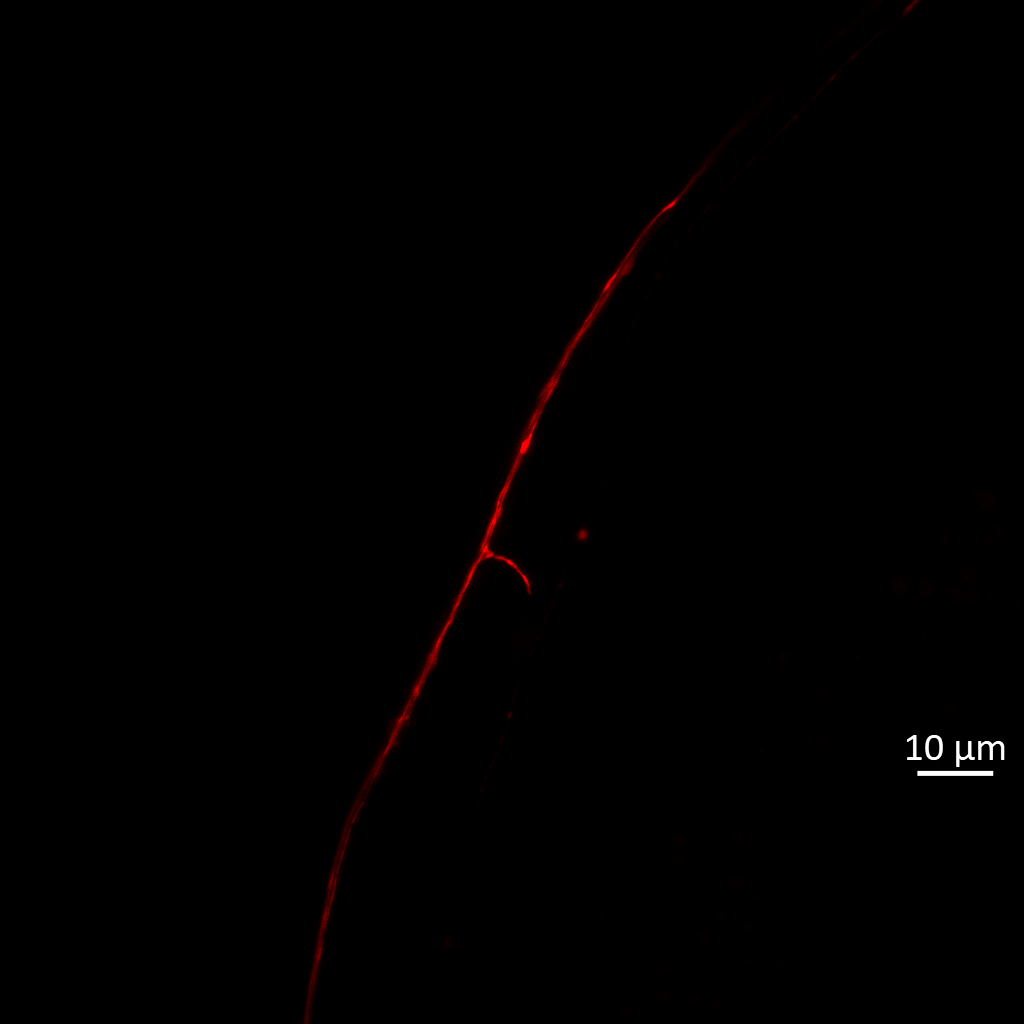

Supplement: Supplementary file 5 — Source data Fig. 2 [file 44319_2025_608_MOESM5_ESM.zip › Figure 2/2F/wing to rod-rod.tif]

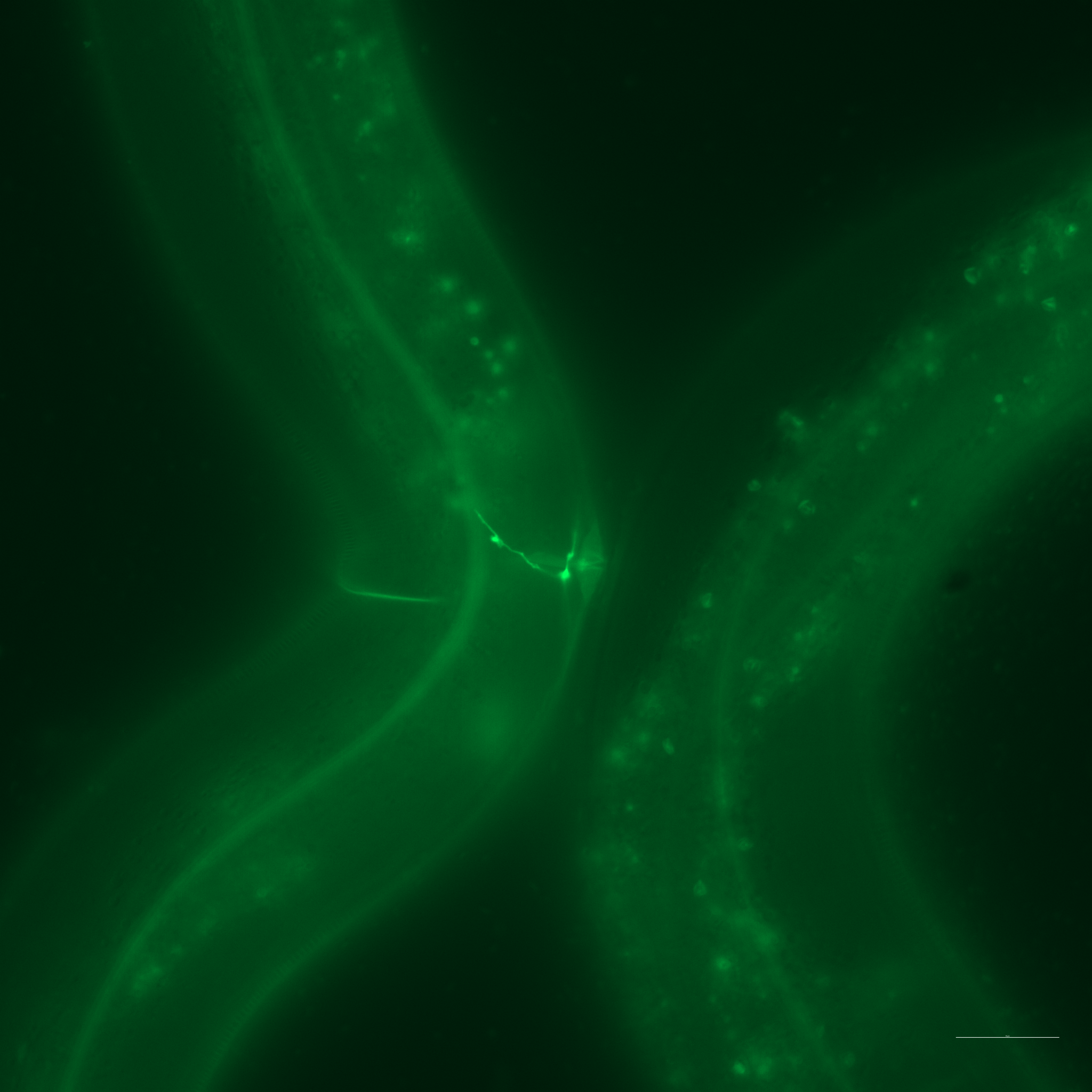

Supplement: Supplementary file 5 — Source data Fig. 2 [file 44319_2025_608_MOESM5_ESM.zip › Figure 2/2F/rod to wing - 60'.tif]

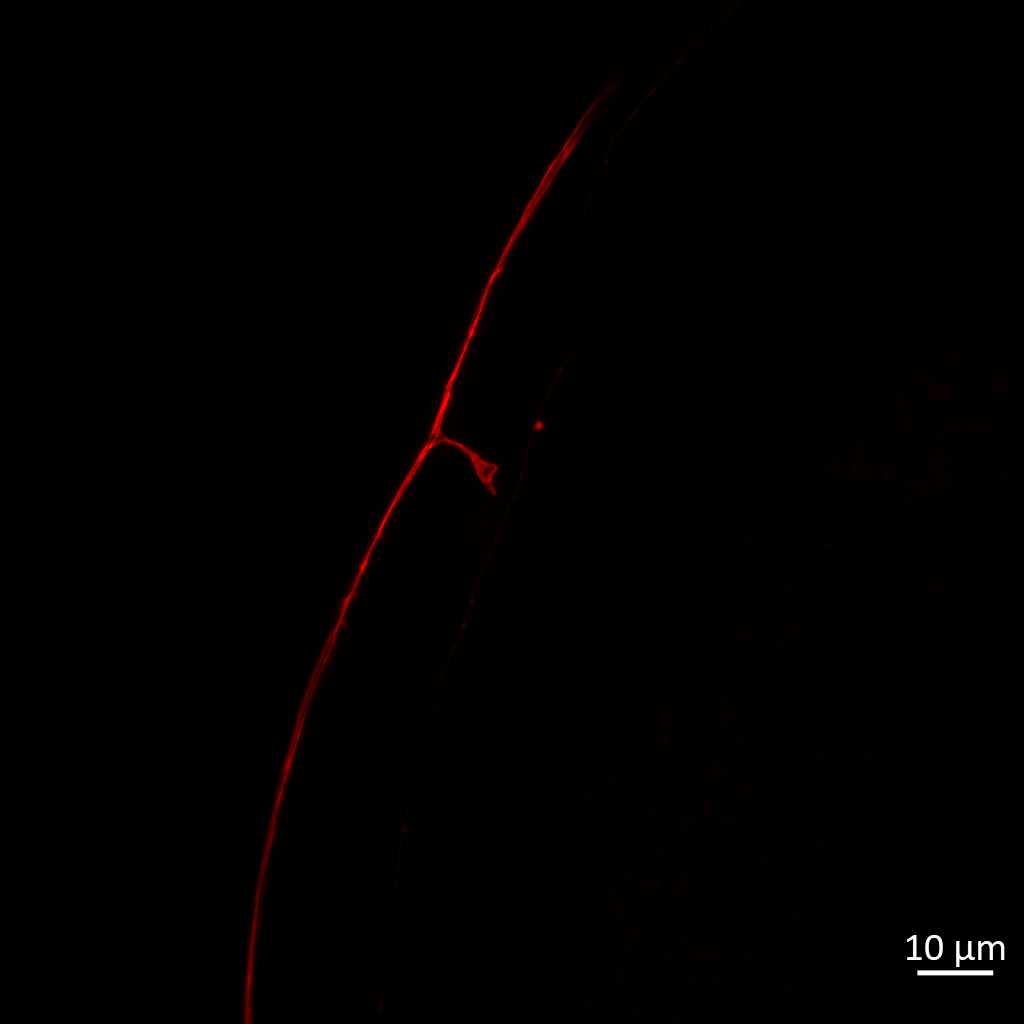

Supplement: Supplementary file 5 — Source data Fig. 2 [file 44319_2025_608_MOESM5_ESM.zip › Figure 2/2F/wing to rod-wing.tif]

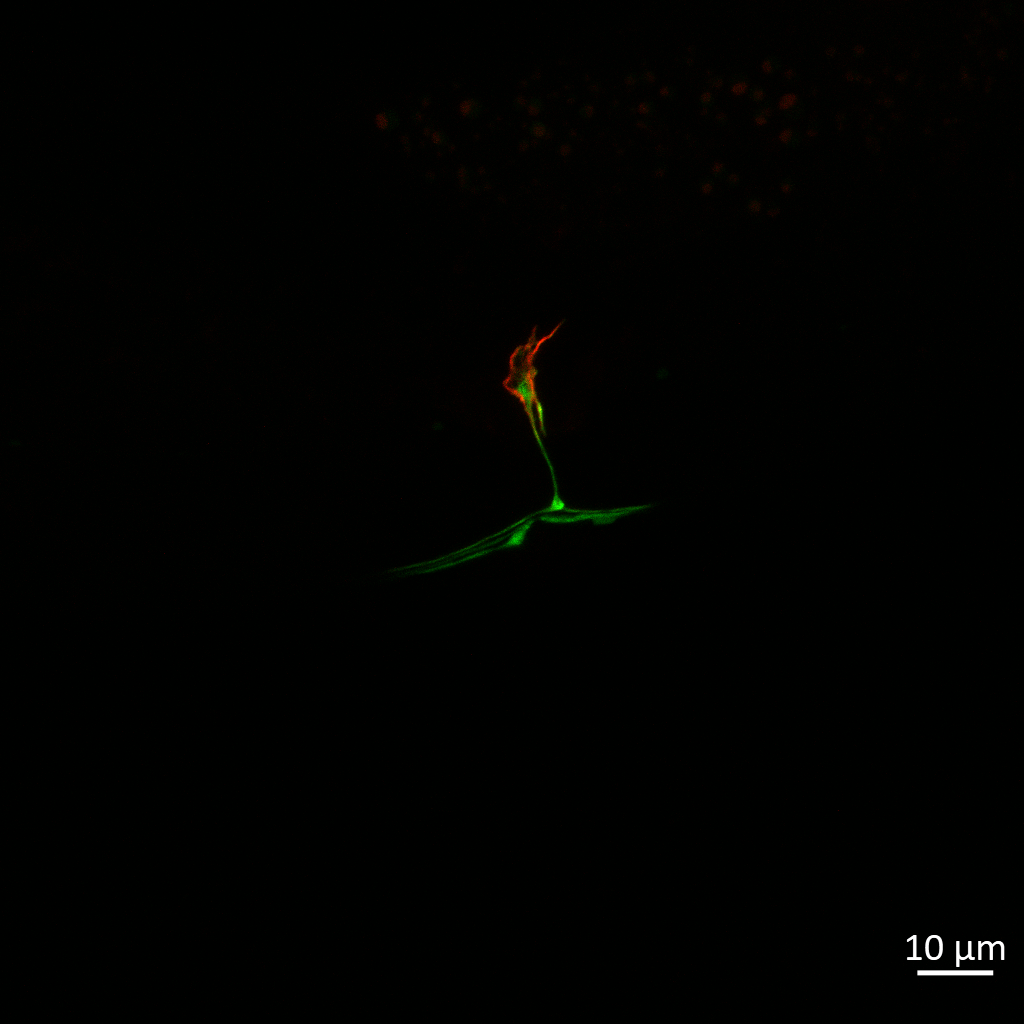

Supplement: Supplementary file 5 — Source data Fig. 2 [file 44319_2025_608_MOESM5_ESM.zip › Figure 2/2D/wing.tif]

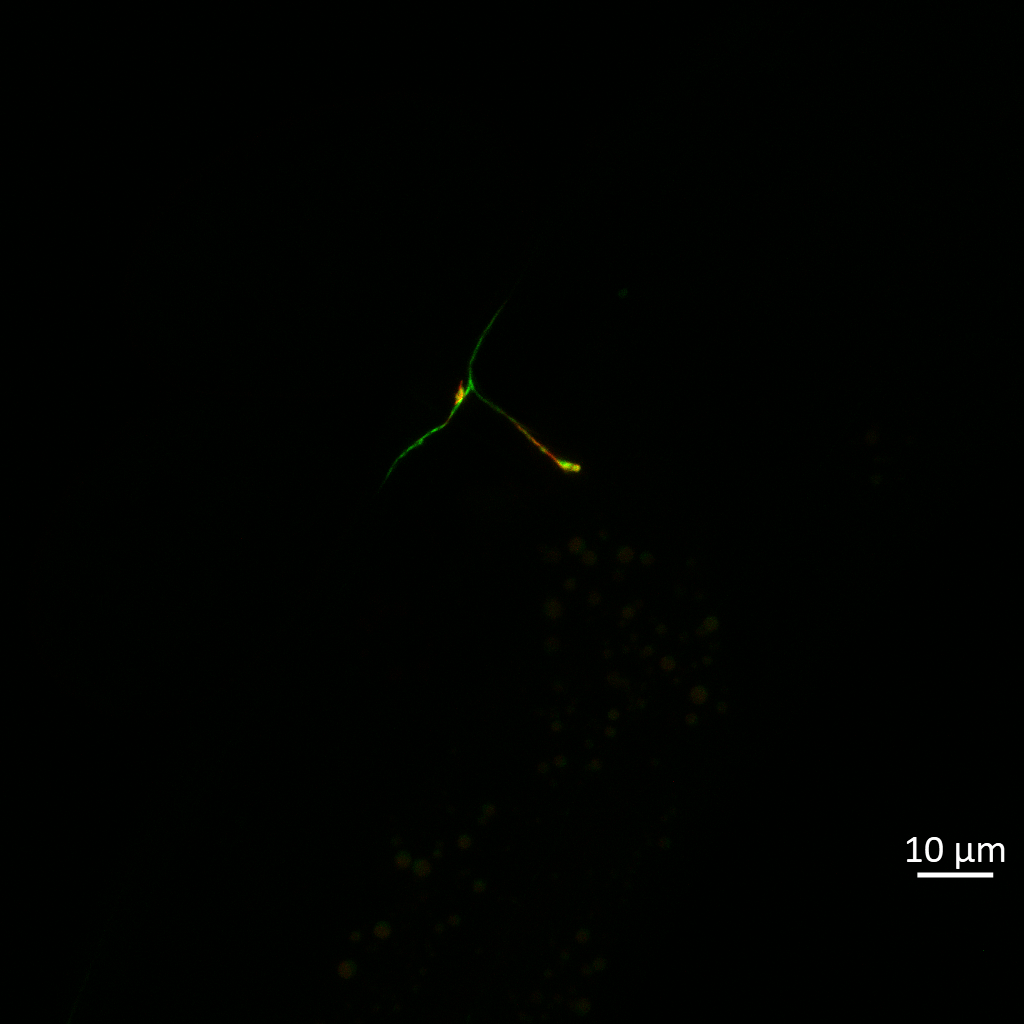

Supplement: Supplementary file 5 — Source data Fig. 2 [file 44319_2025_608_MOESM5_ESM.zip › Figure 2/2D/rod.tif]

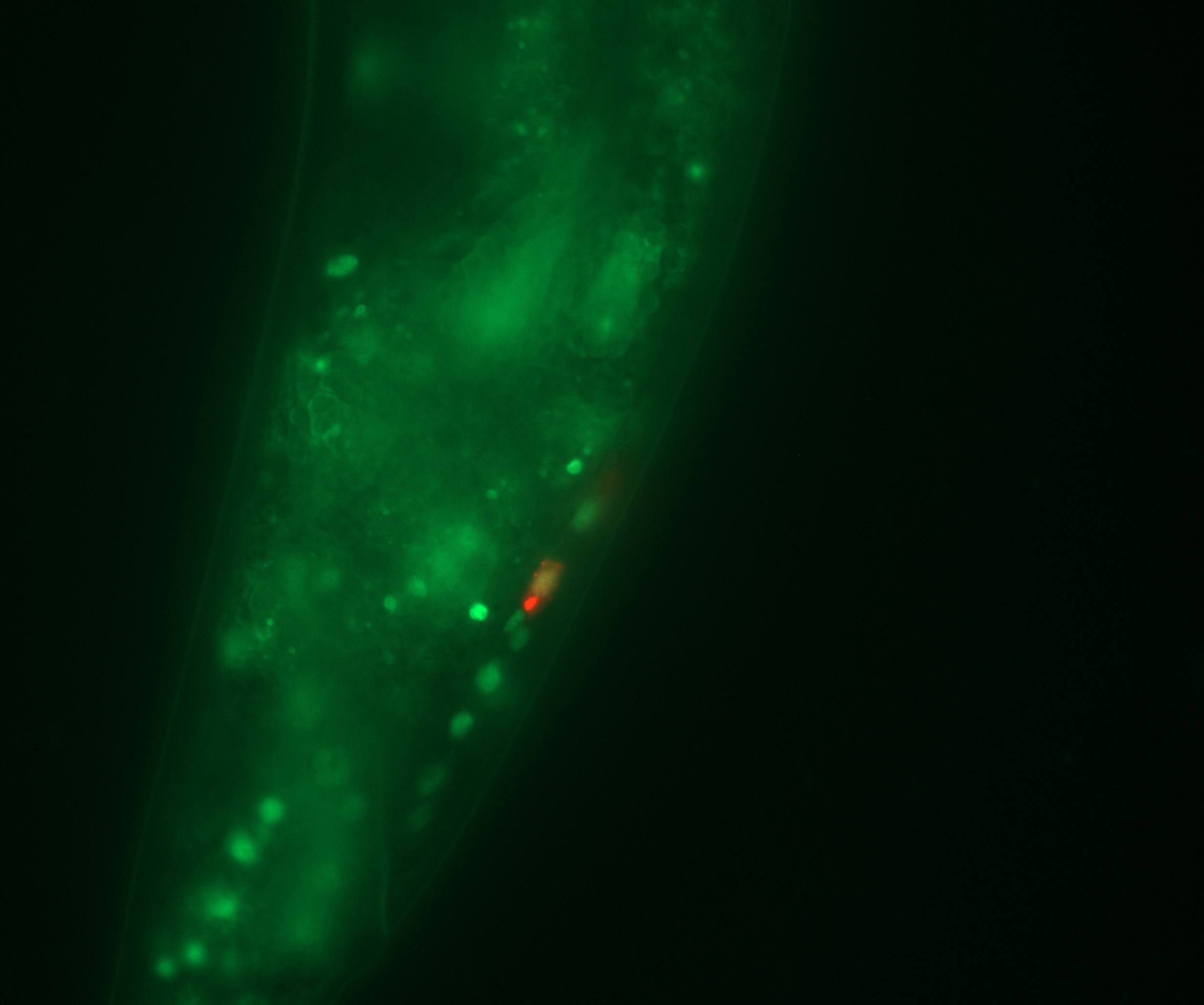

Supplement: Supplementary file 7 — Source data Fig. 4 [file 44319_2025_608_MOESM7_ESM.zip › Figure 4/4B/PVP Pocr-3 mCherry herm.jpg]

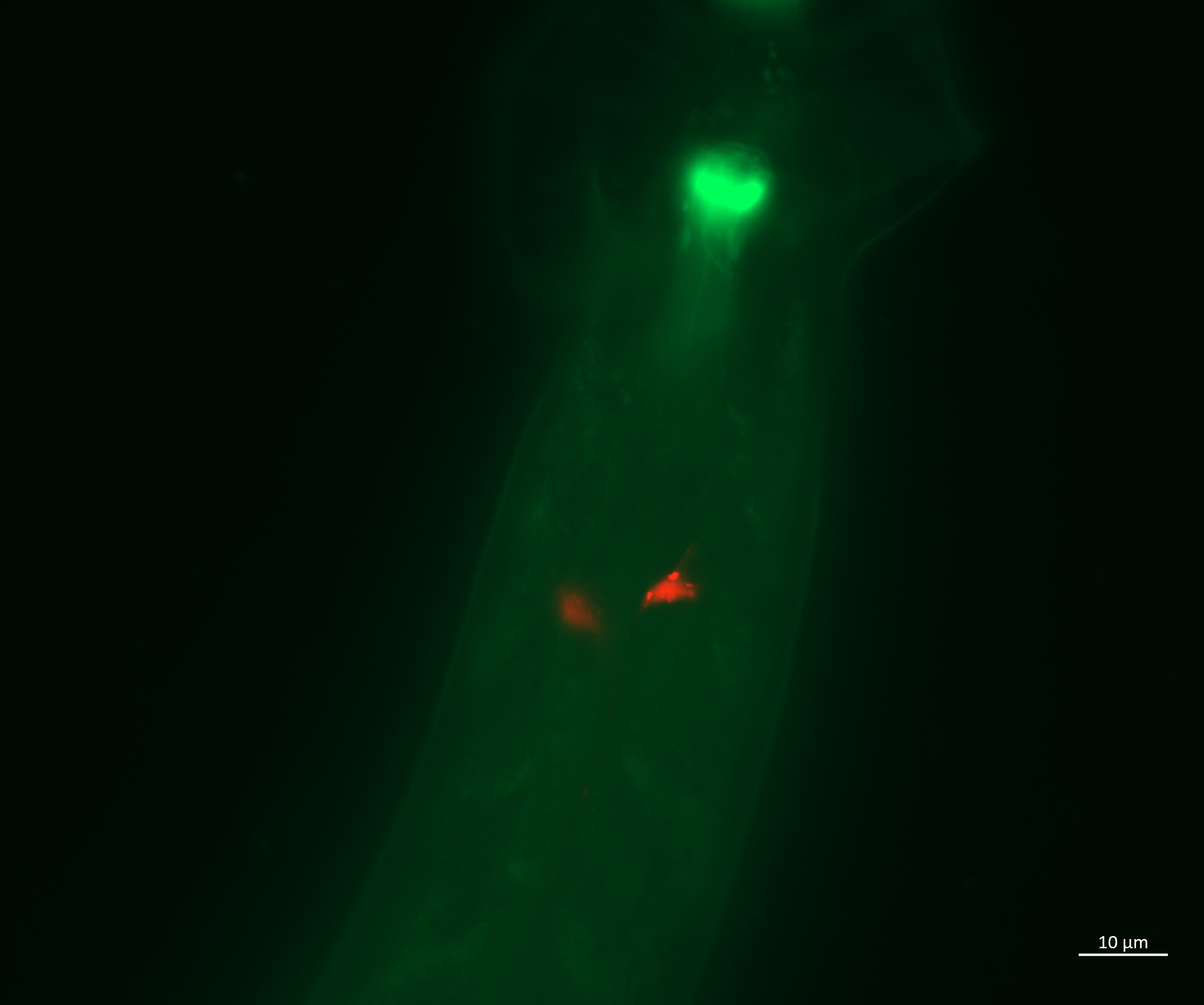

Supplement: Supplementary file 7 — Source data Fig. 4 [file 44319_2025_608_MOESM7_ESM.zip › Figure 4/4B/PVP Pocr-3 mCherry male.jpg]

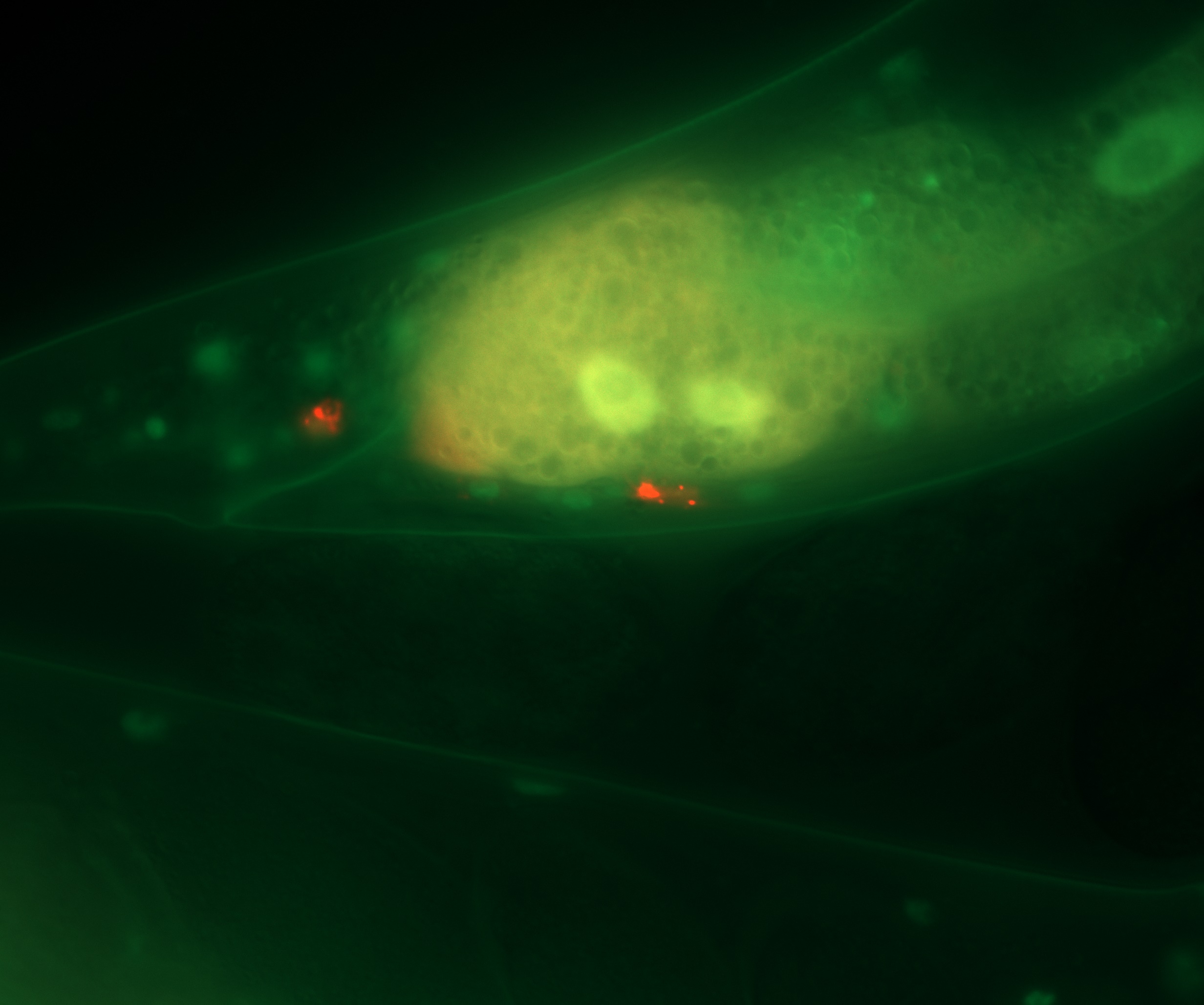

Supplement: Supplementary file 7 — Source data Fig. 4 [file 44319_2025_608_MOESM7_ESM.zip › Figure 4/4B/PVP Pocr-3 FEM-3 herm.jpg]

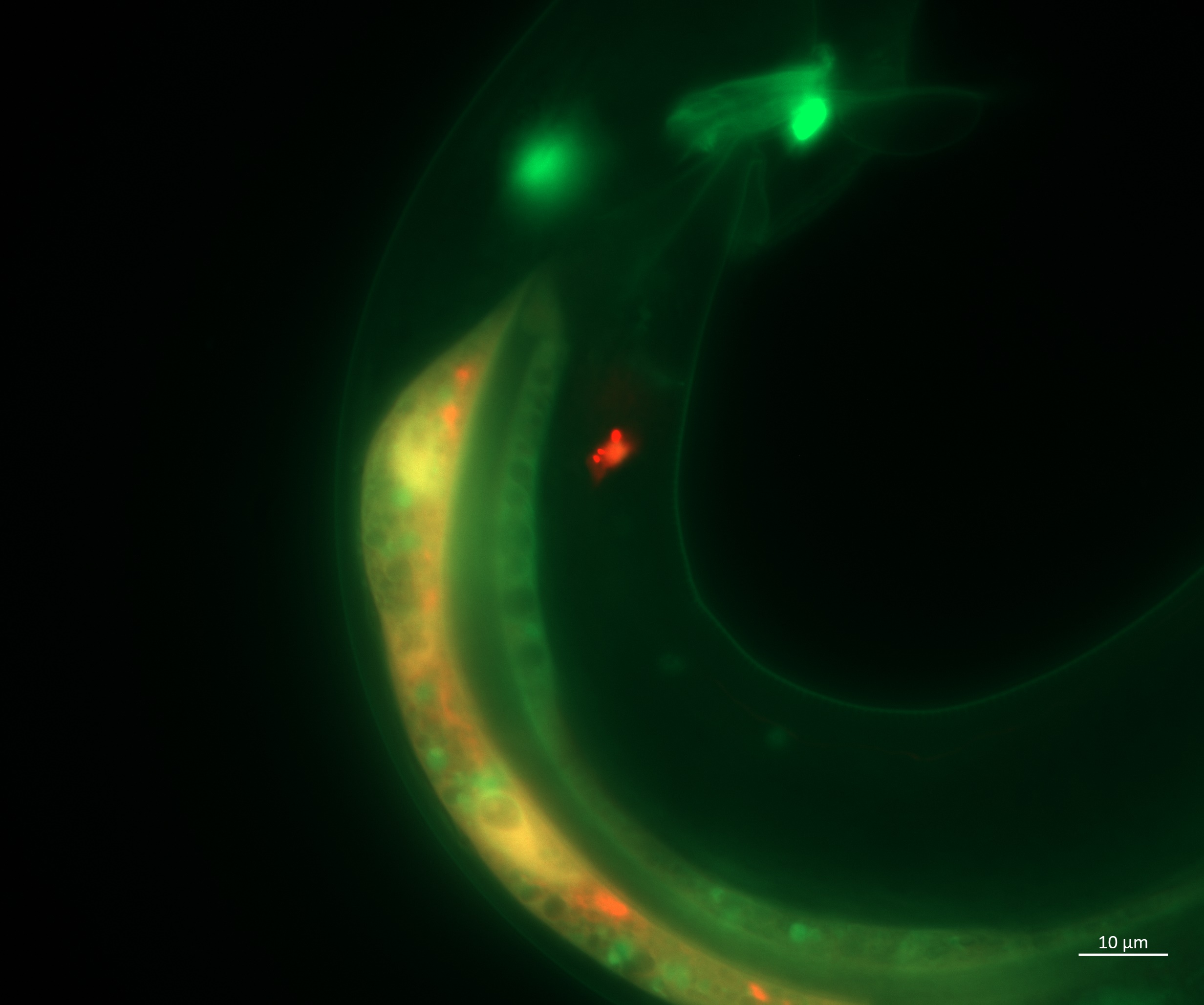

Supplement: Supplementary file 7 — Source data Fig. 4 [file 44319_2025_608_MOESM7_ESM.zip › Figure 4/4B/PVP Pocr-3 TRA-2 male.jpg]

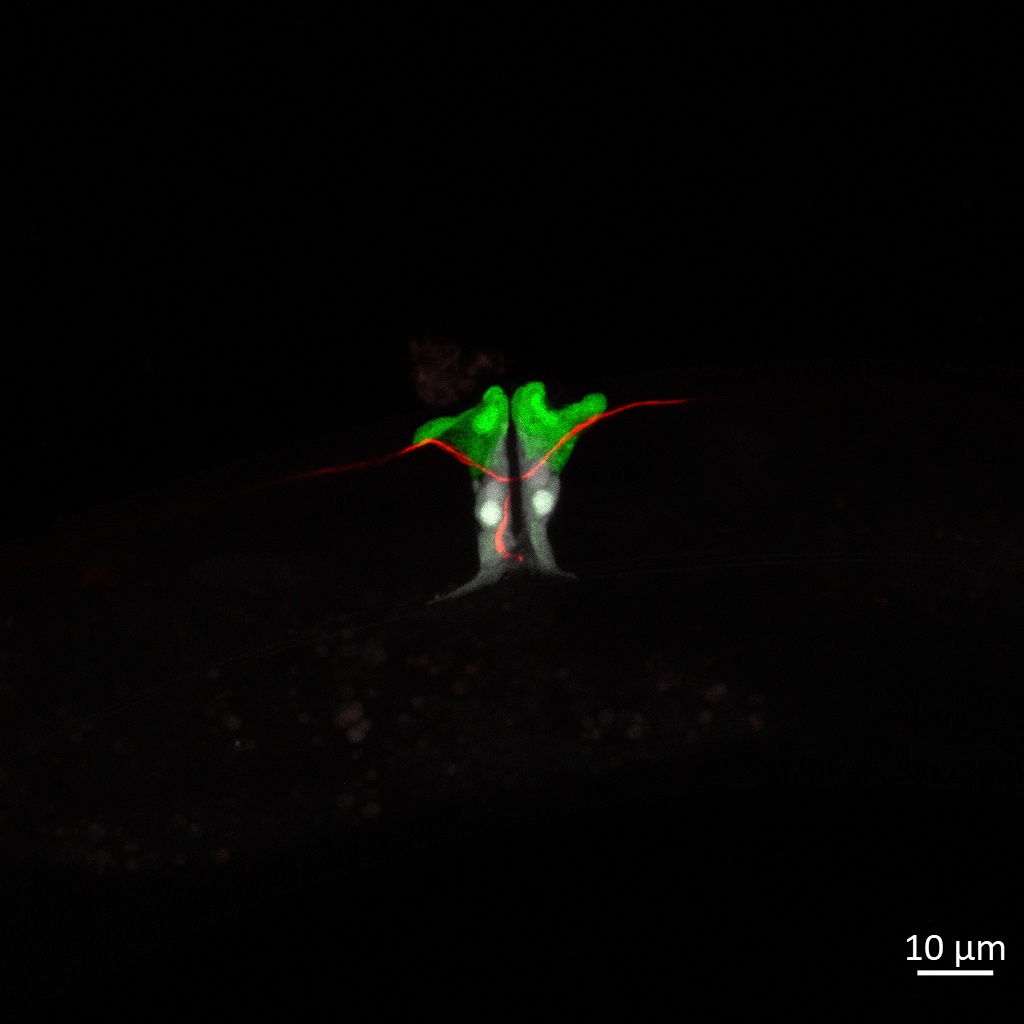

Supplement: Supplementary file 8 — Source data Fig. 5 [file 44319_2025_608_MOESM8_ESM.zip › Figure 5/5C/vulva cell + PVP.jpg]

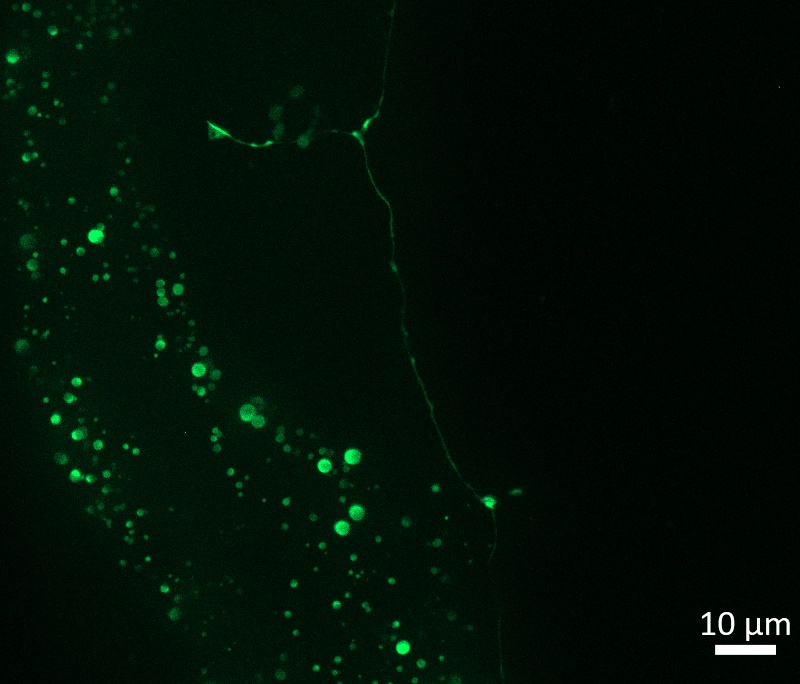

Supplement: Supplementary file 8 — Source data Fig. 5 [file 44319_2025_608_MOESM8_ESM.zip › Figure 5/5E/let-60 herm GFP.jpg]

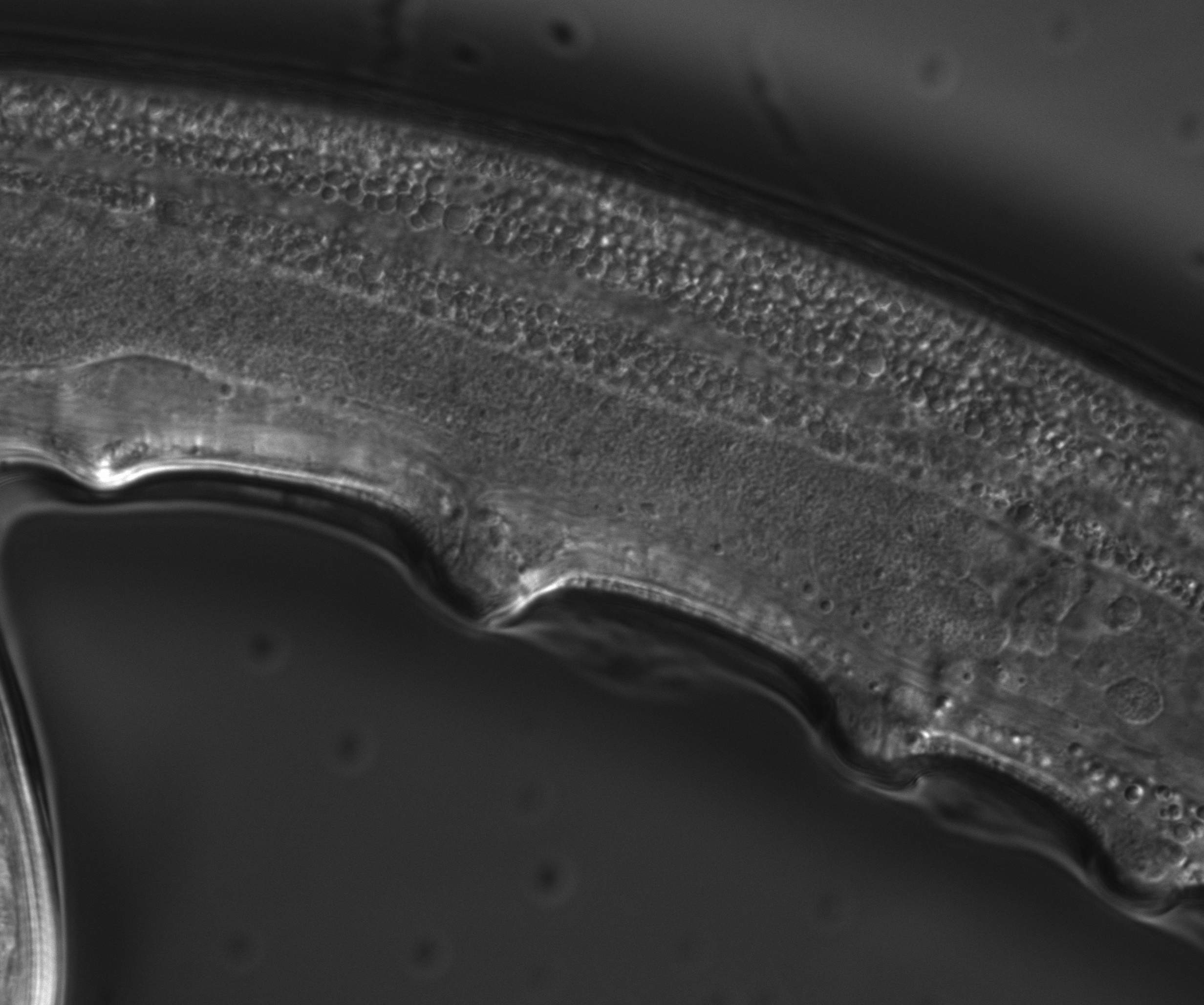

Supplement: Supplementary file 8 — Source data Fig. 5 [file 44319_2025_608_MOESM8_ESM.zip › Figure 5/5E/lin-12 male DIC.jpg]

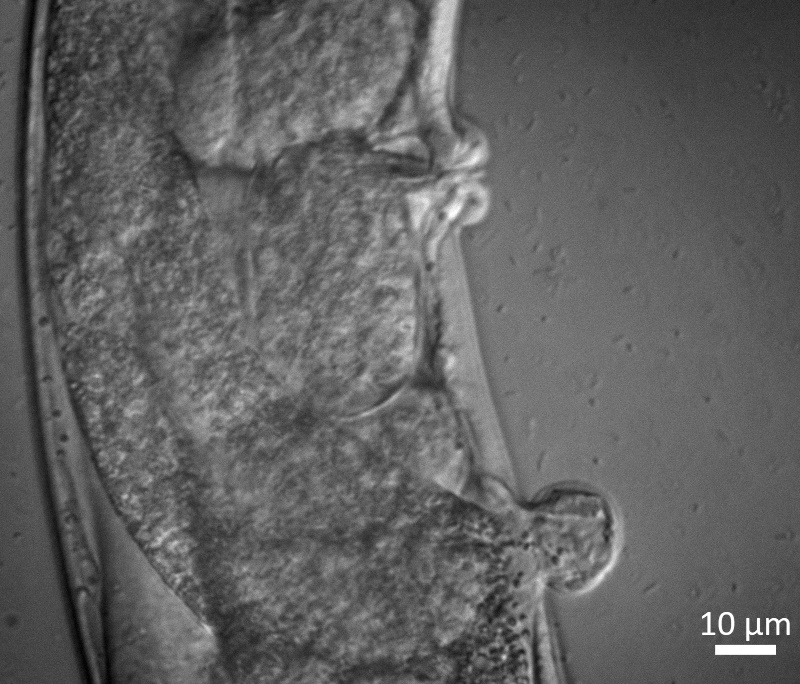

Supplement: Supplementary file 8 — Source data Fig. 5 [file 44319_2025_608_MOESM8_ESM.zip › Figure 5/5E/let-60 herm DIC.jpg]

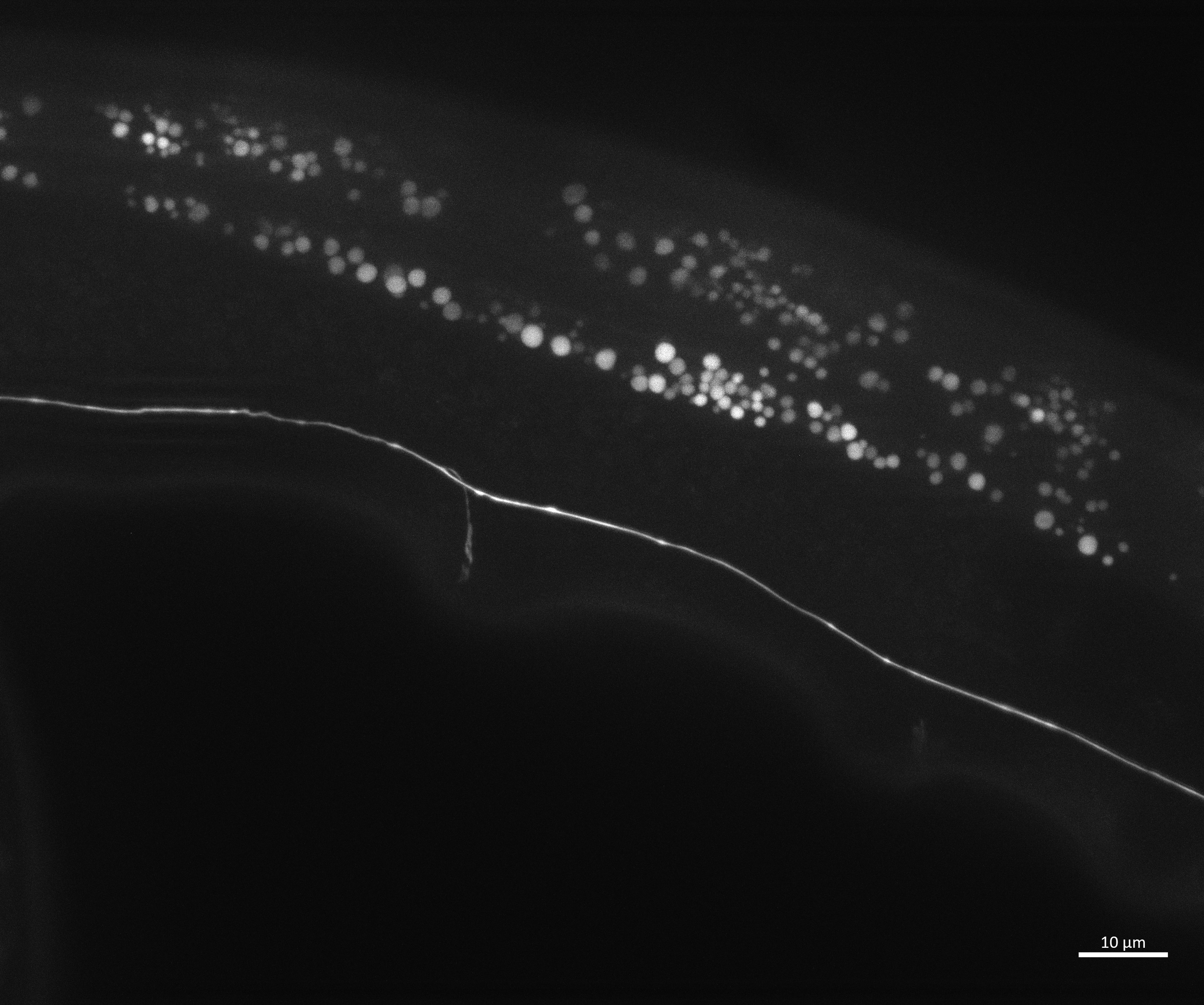

Supplement: Supplementary file 8 — Source data Fig. 5 [file 44319_2025_608_MOESM8_ESM.zip › Figure 5/5E/lin-12 male GFP.jpg]

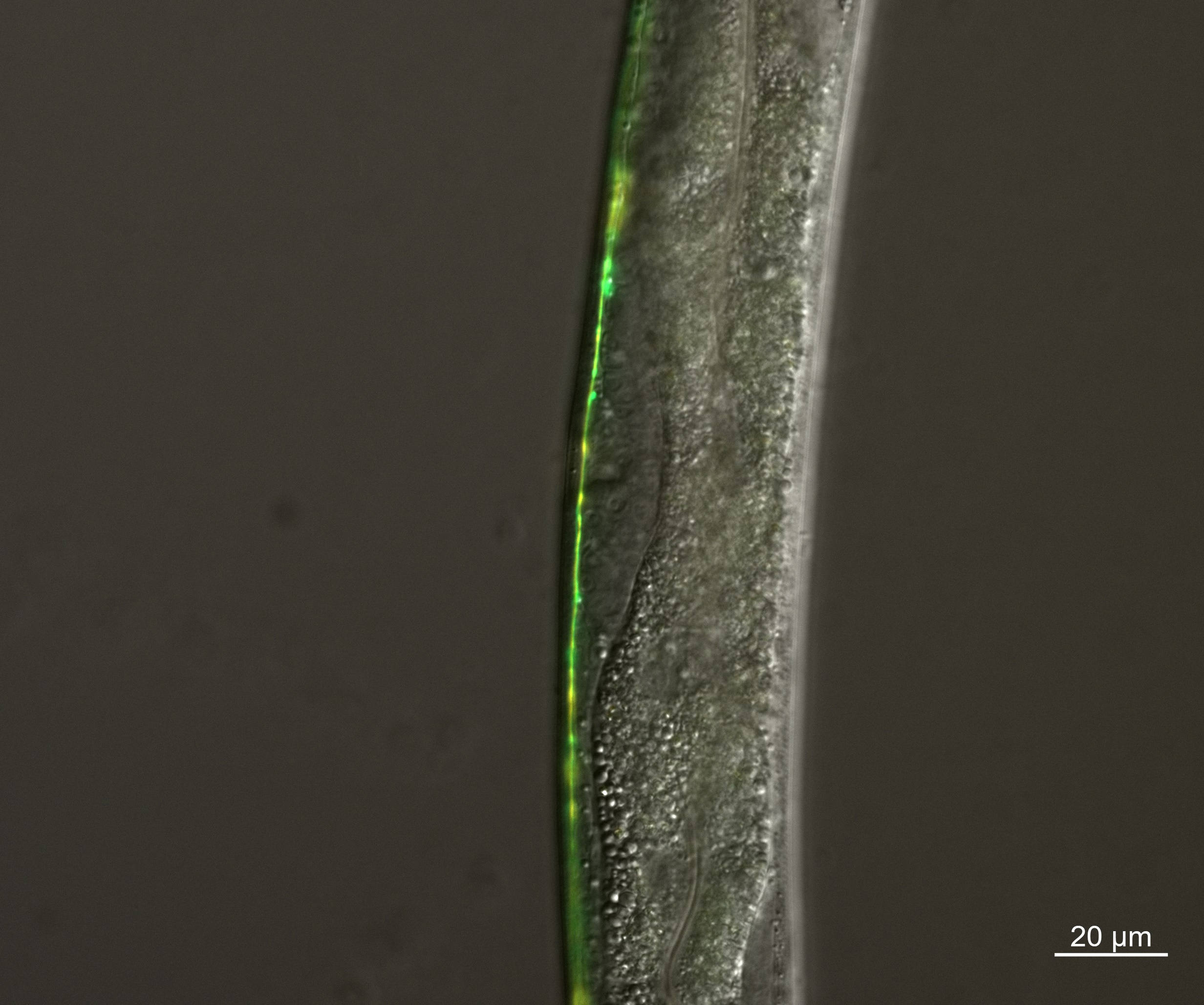

Supplement: Supplementary file 9 — Source data Fig. 6 [file 44319_2025_608_MOESM9_ESM.zip › Figure 6/6B/daf-2 L4 Factin.jpg]

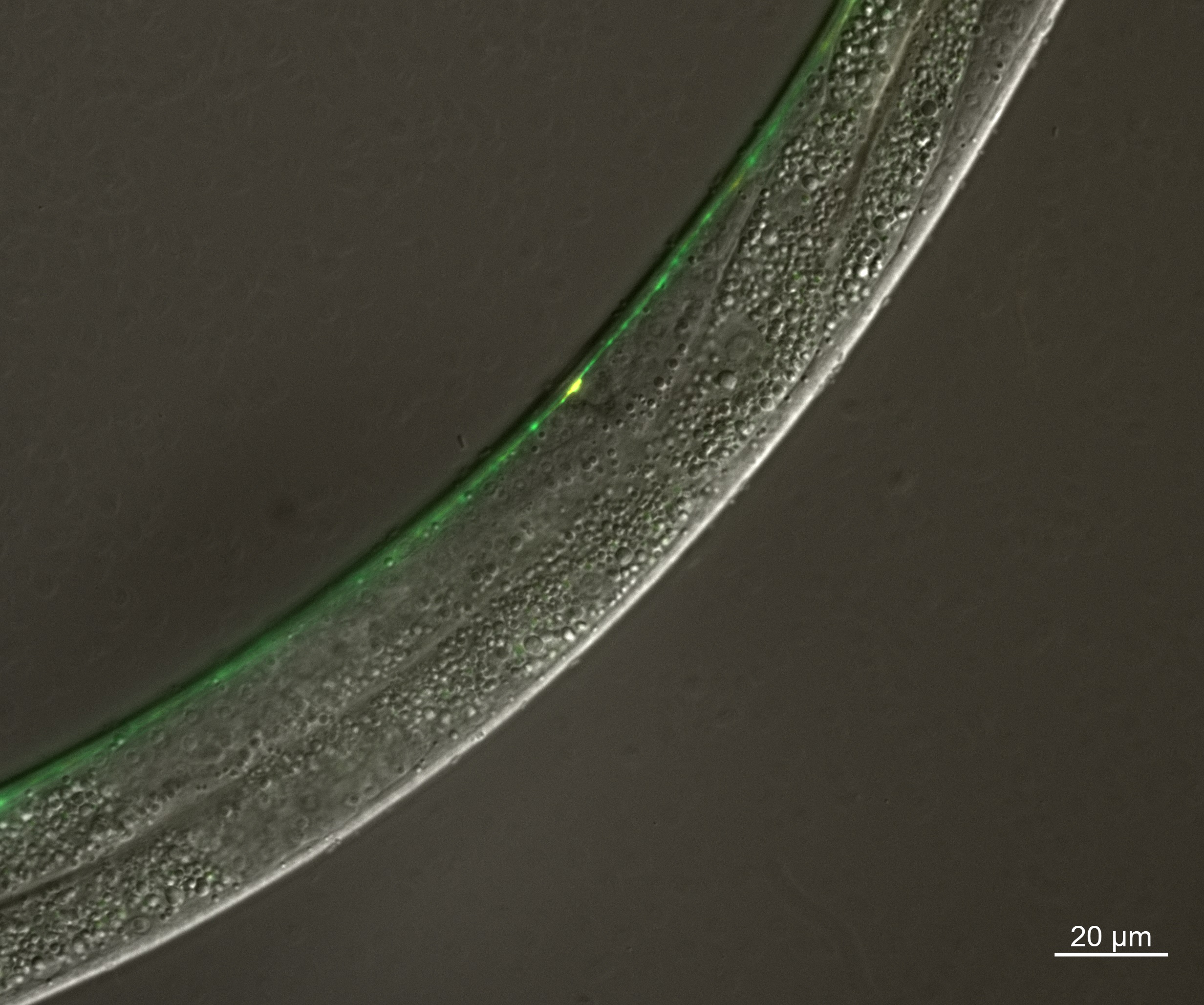

Supplement: Supplementary file 9 — Source data Fig. 6 [file 44319_2025_608_MOESM9_ESM.zip › Figure 6/6B/WT L4 Factin.jpg]

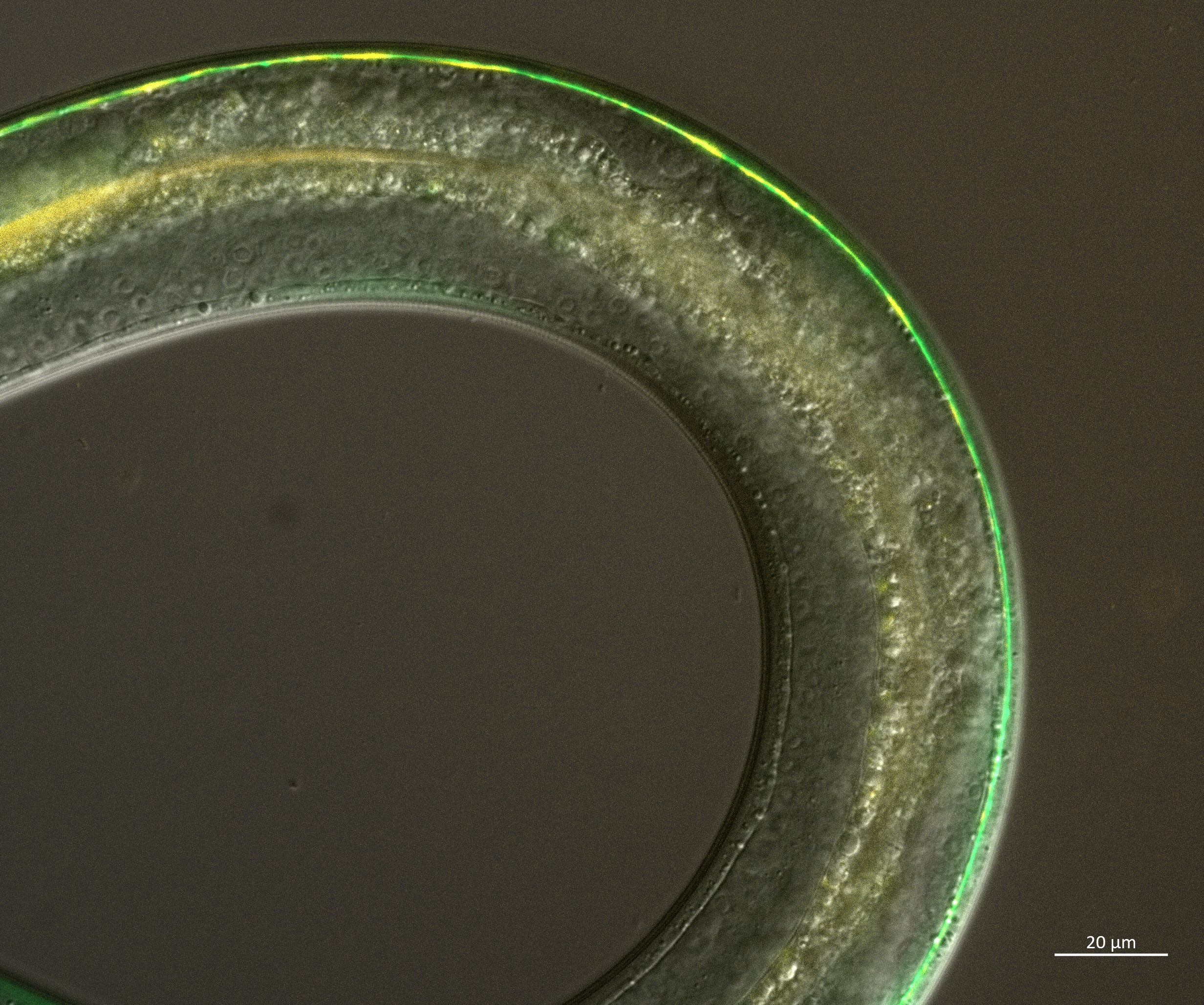

Supplement: Supplementary file 9 — Source data Fig. 6 [file 44319_2025_608_MOESM9_ESM.zip › Figure 6/6B/lin-39 L4 Factin.jpg]

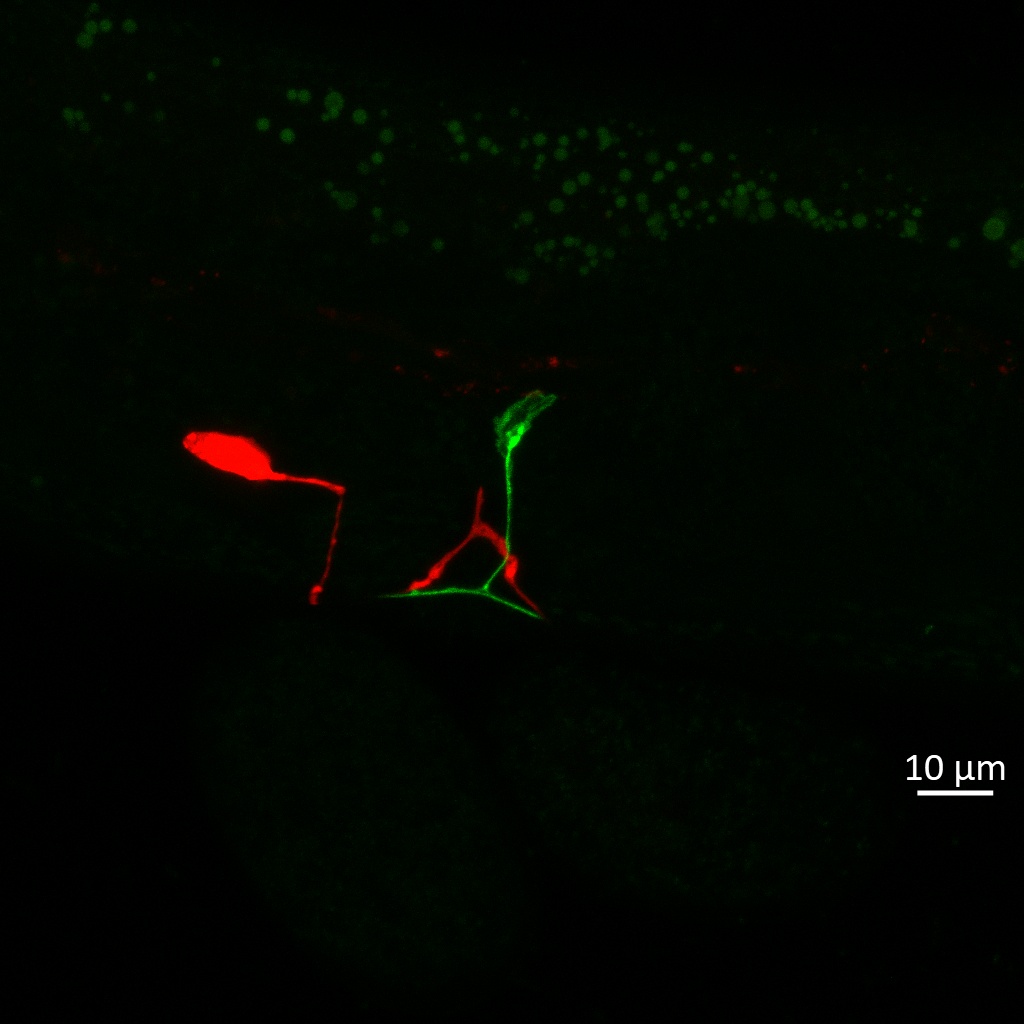

Supplement: Supplementary file 10 — Source data Fig. 7 [file 44319_2025_608_MOESM10_ESM.zip › Figure 7/7C/HSN.jpg]

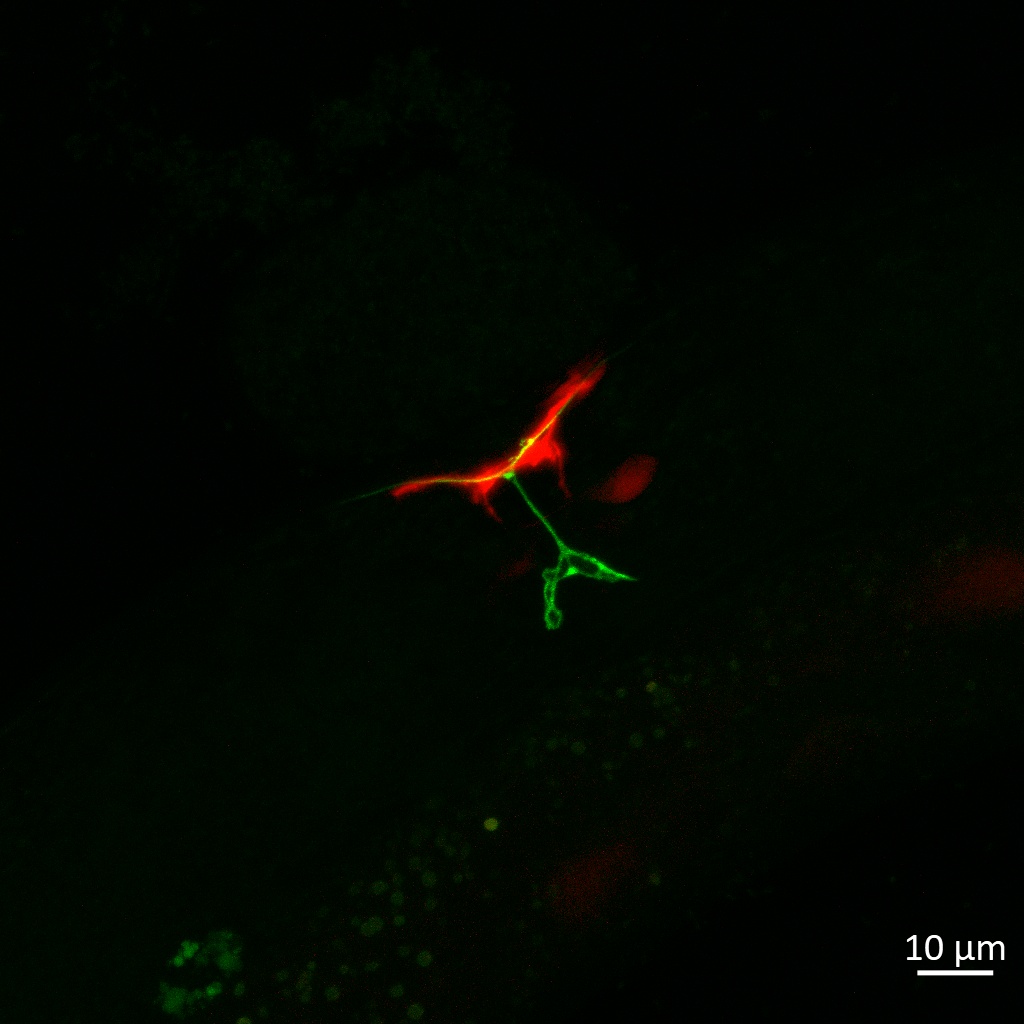

Supplement: Supplementary file 10 — Source data Fig. 7 [file 44319_2025_608_MOESM10_ESM.zip › Figure 7/7C/VC.jpg]

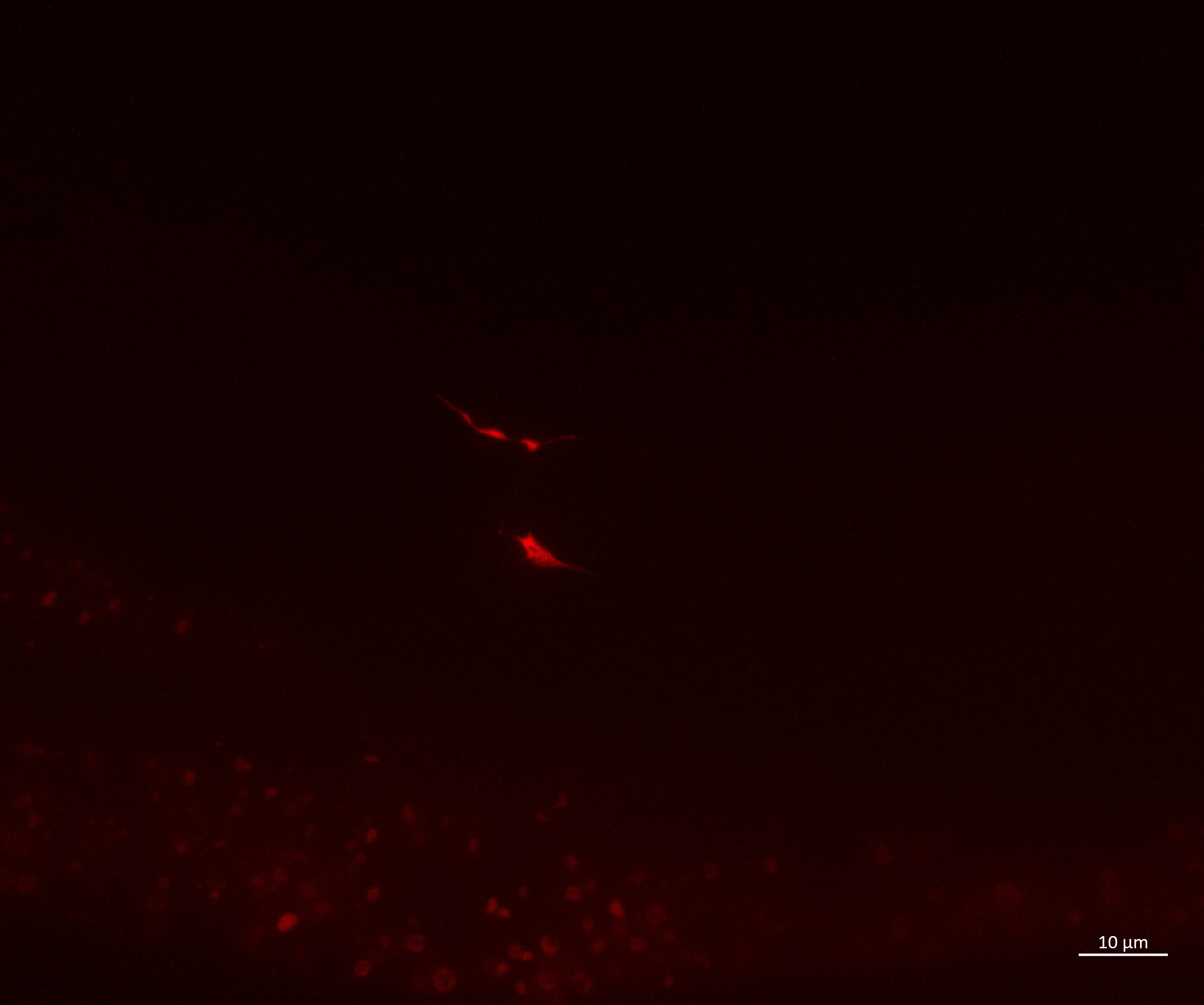

Supplement: Supplementary file 11 — Appendix and EV Figures Source Data [file 44319_2025_608_MOESM11_ESM.zip › Figure EV1/EV1A/branch Ppdf-1.tif]

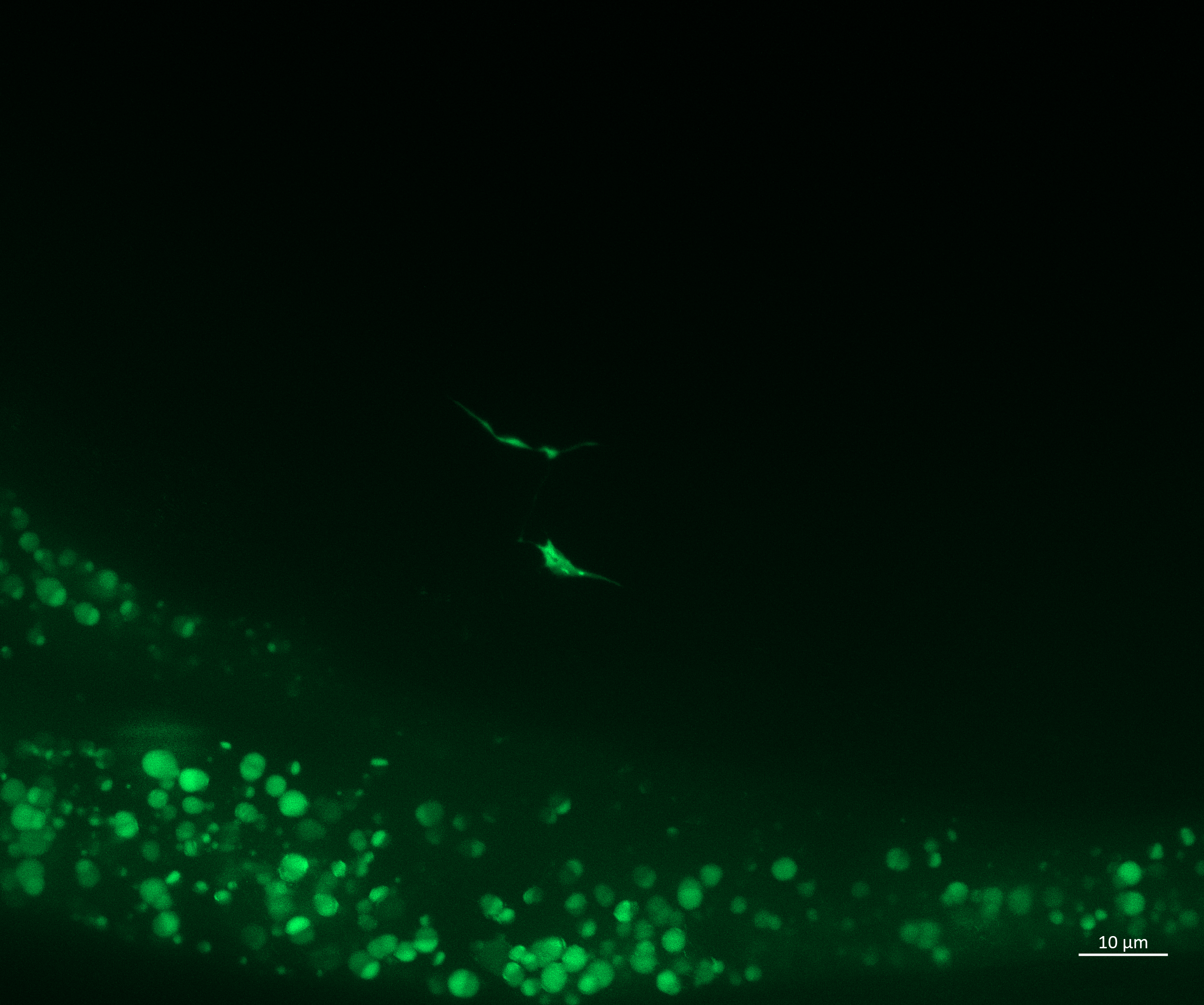

Supplement: Supplementary file 11 — Appendix and EV Figures Source Data [file 44319_2025_608_MOESM11_ESM.zip › Figure EV1/EV1A/branch Pocr-3.tif]

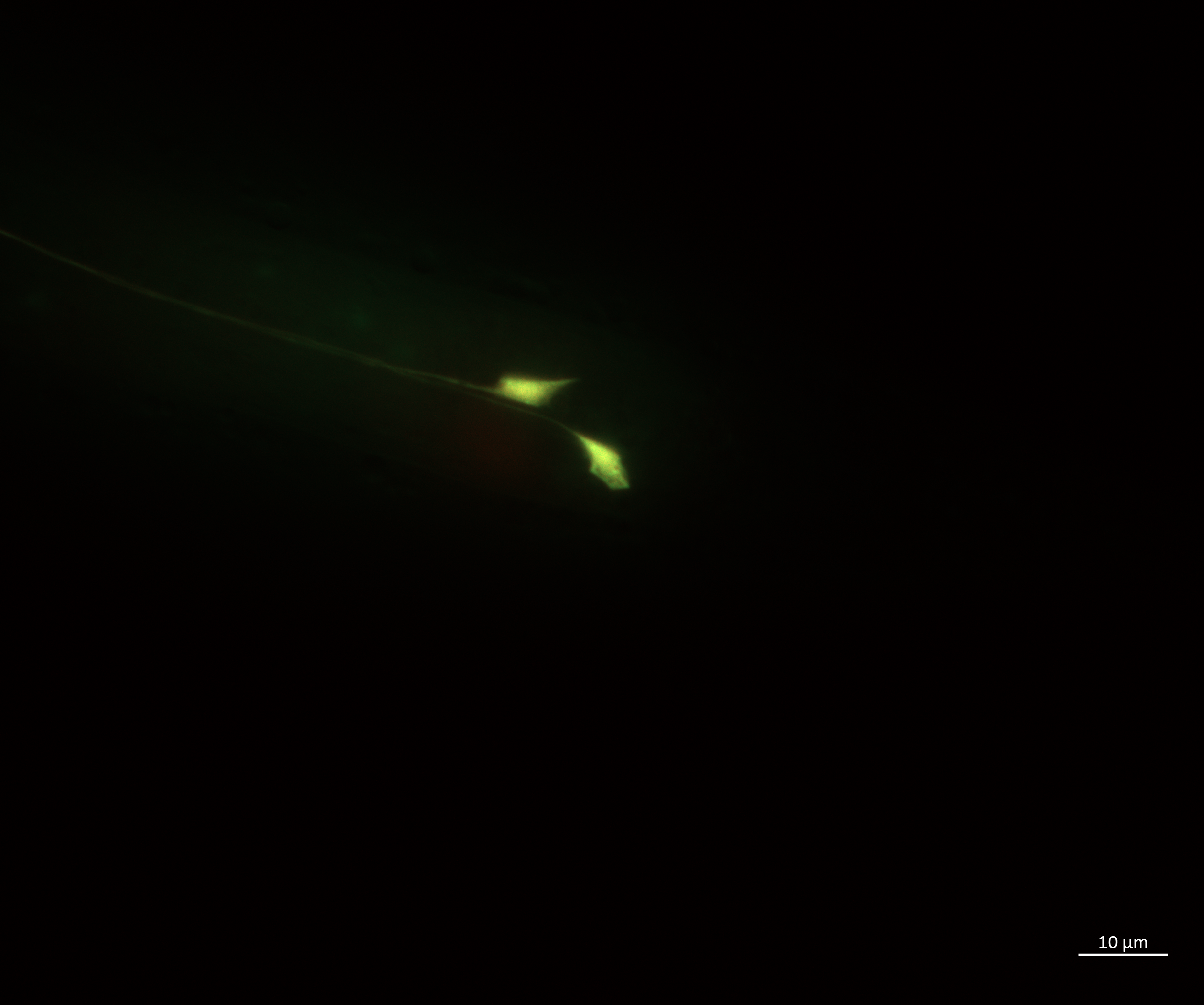

Supplement: Supplementary file 11 — Appendix and EV Figures Source Data [file 44319_2025_608_MOESM11_ESM.zip › Figure EV1/EV1A/soma.tif]

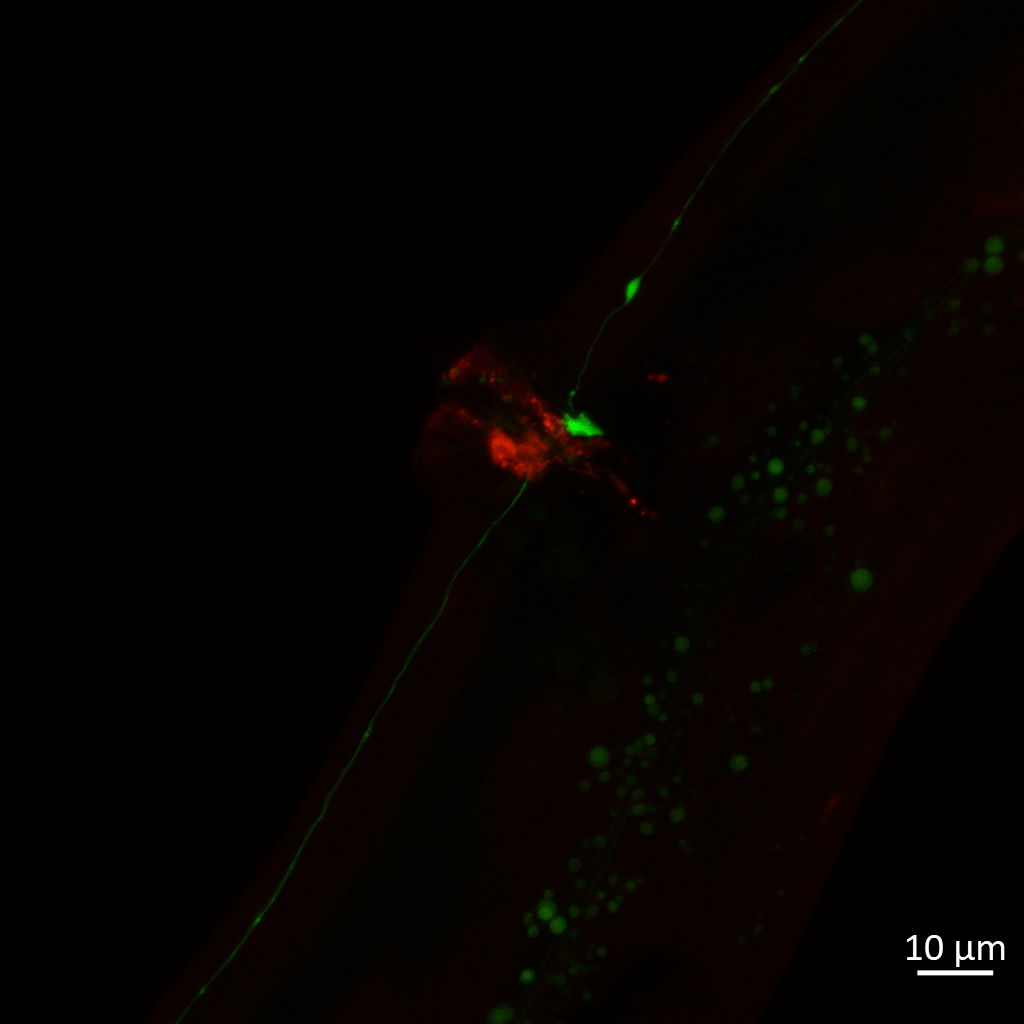

Supplement: Supplementary file 11 — Appendix and EV Figures Source Data [file 44319_2025_608_MOESM11_ESM.zip › Appendix figure S3/daf-6 killed.tif]

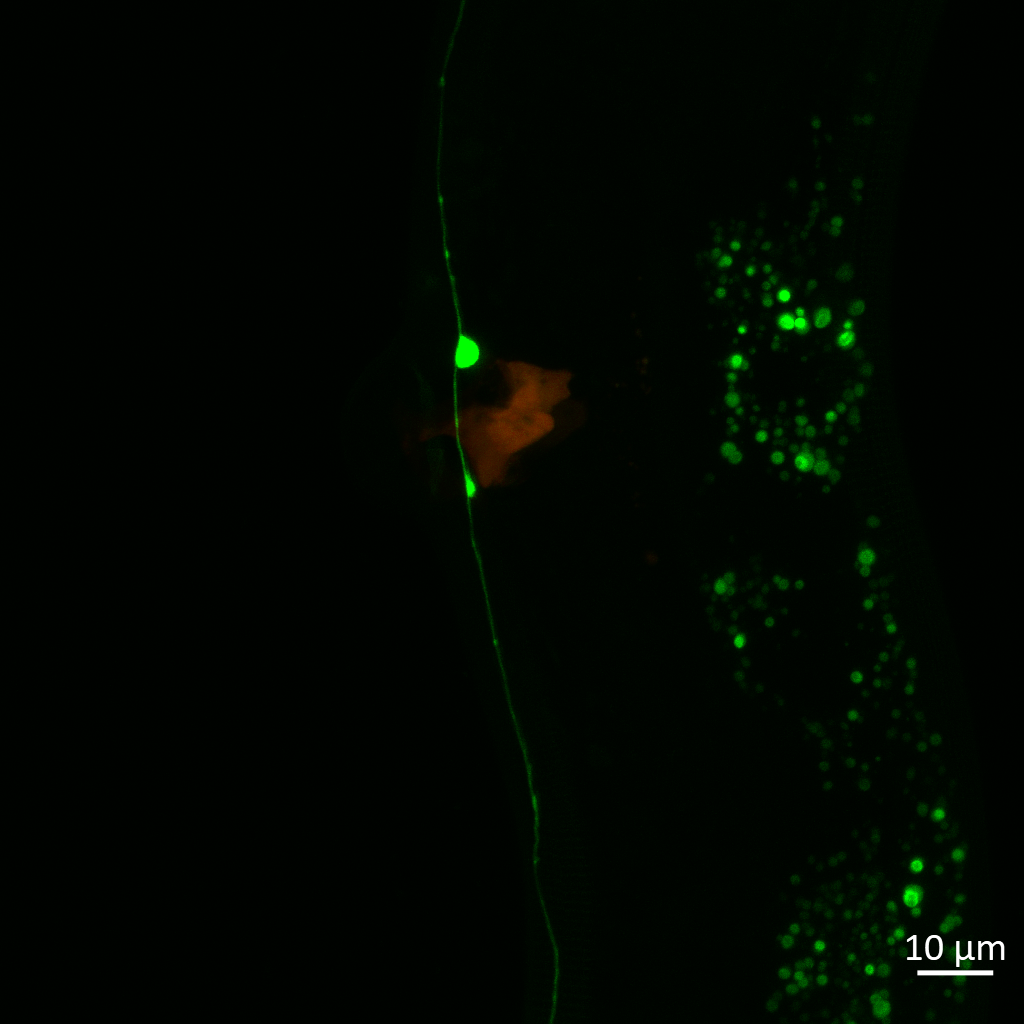

Supplement: Supplementary file 11 — Appendix and EV Figures Source Data [file 44319_2025_608_MOESM11_ESM.zip › Appendix figure S3/cdh-3 killed.tif]

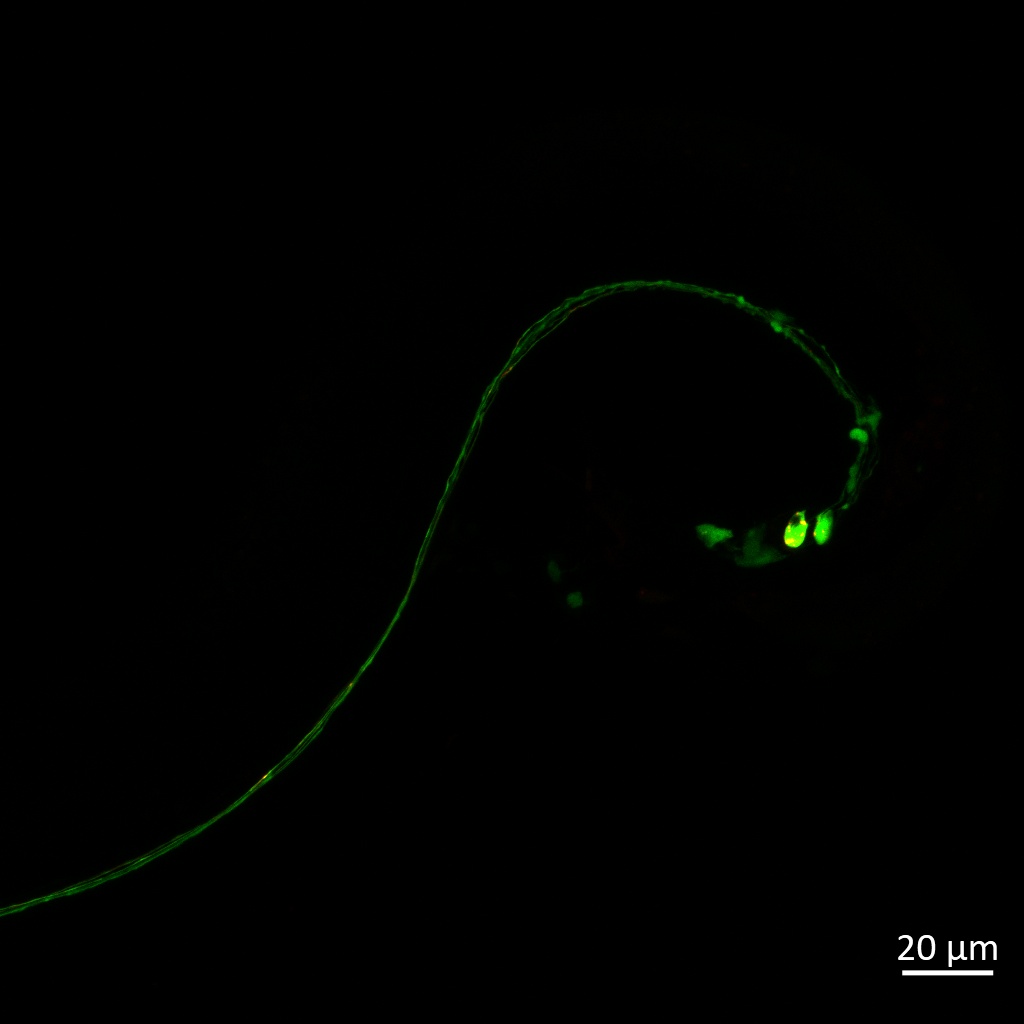

Supplement: Supplementary file 11 — Appendix and EV Figures Source Data [file 44319_2025_608_MOESM11_ESM.zip › Appendix figure S2/LifeAct male Soma.jpg]

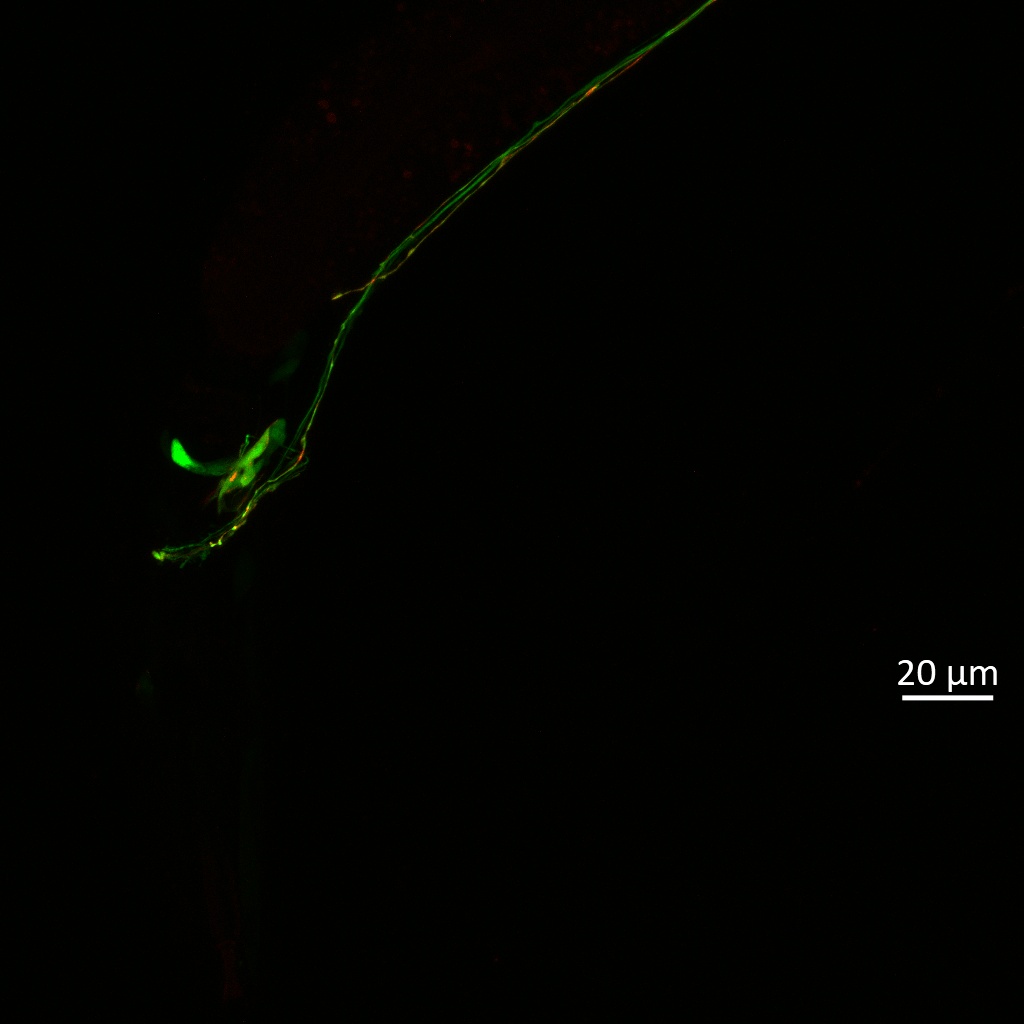

Supplement: Supplementary file 11 — Appendix and EV Figures Source Data [file 44319_2025_608_MOESM11_ESM.zip › Appendix figure S2/LifeAct male Nerve ring.jpg]

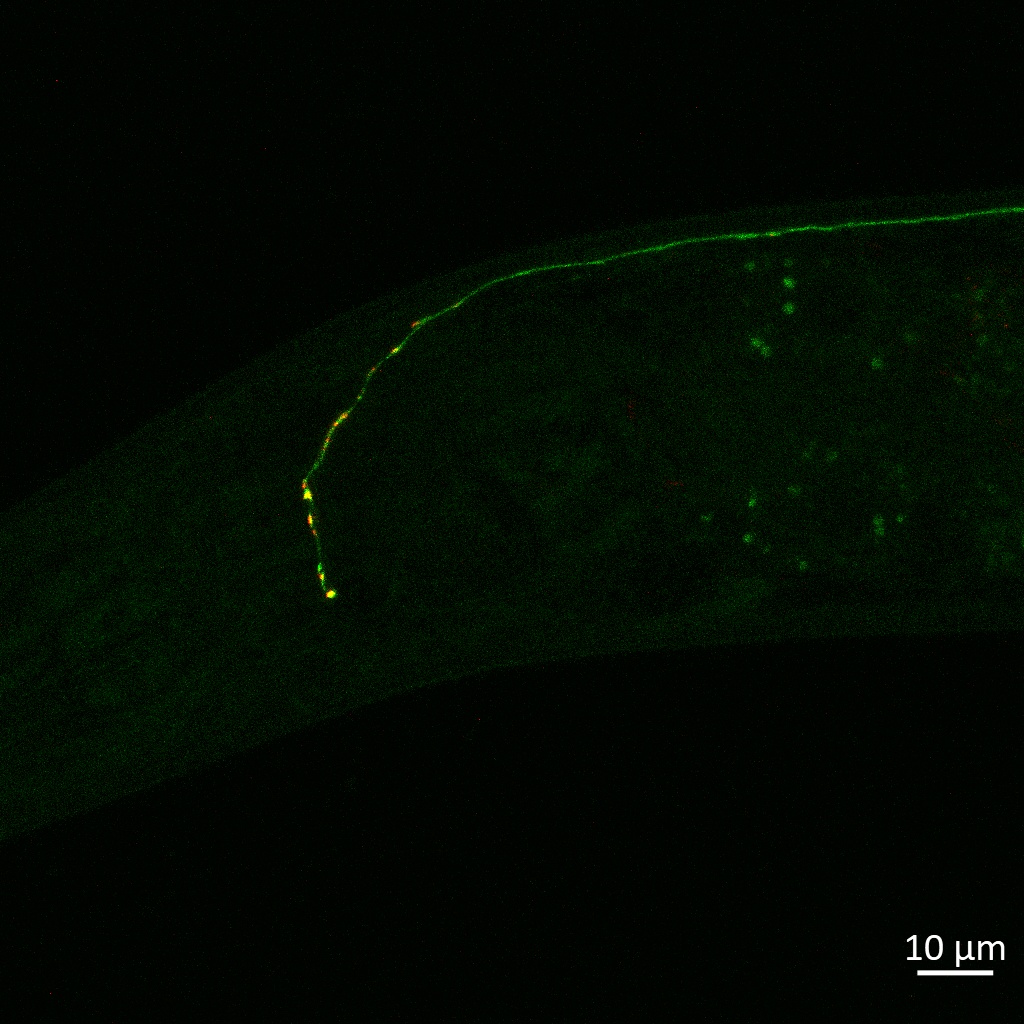

Supplement: Supplementary file 11 — Appendix and EV Figures Source Data [file 44319_2025_608_MOESM11_ESM.zip › Appendix figure S1/S1A/RAB3 nerve ring.jpg]

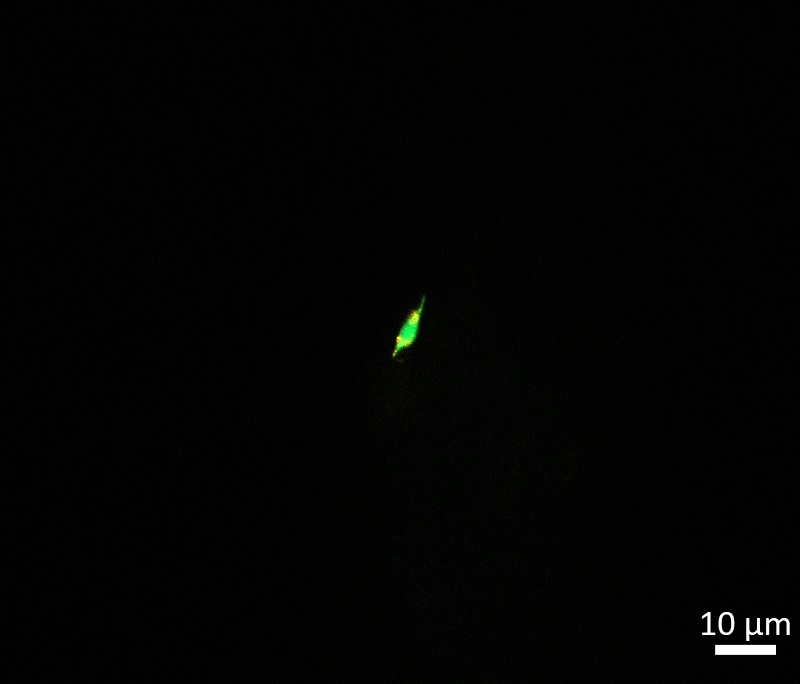

Supplement: Supplementary file 11 — Appendix and EV Figures Source Data [file 44319_2025_608_MOESM11_ESM.zip › Appendix figure S1/S1C/KAP-1.jpg]

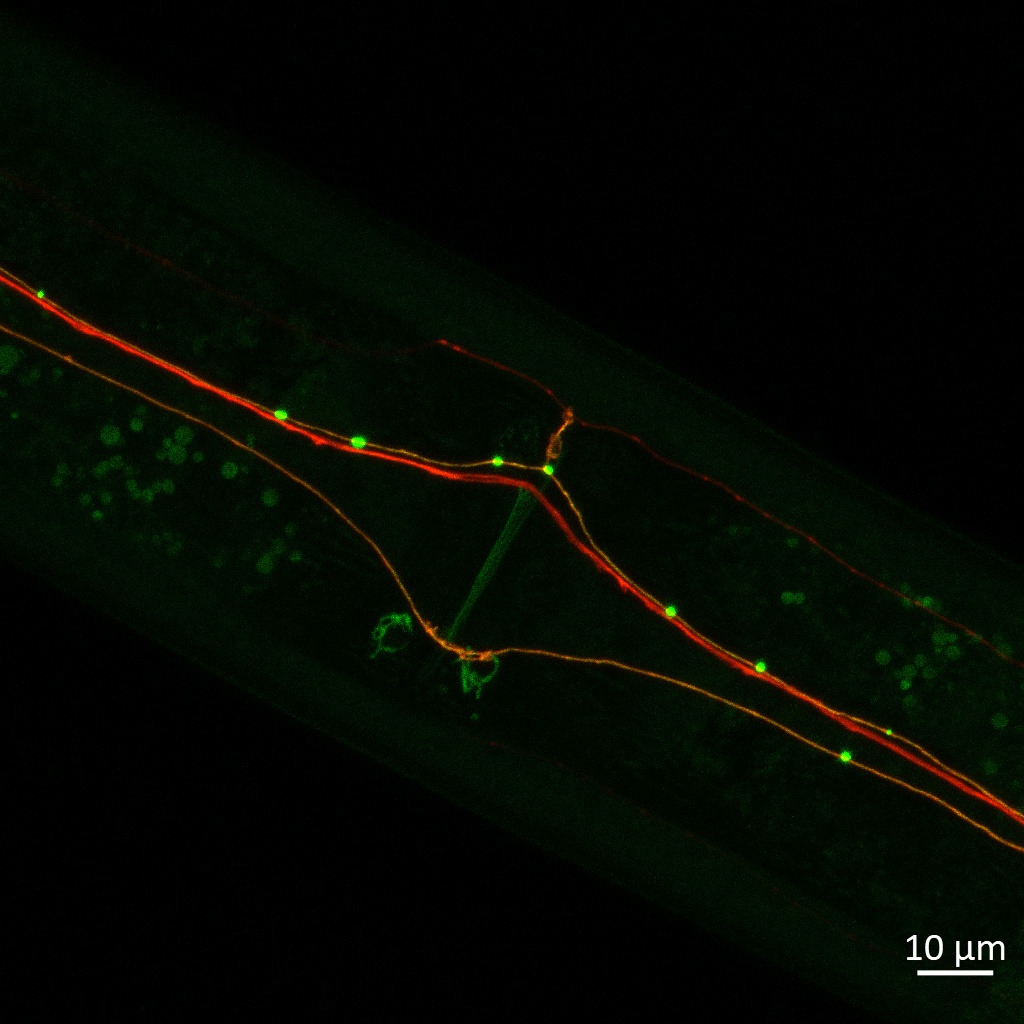

Supplement: Supplementary file 11 — Appendix and EV Figures Source Data [file 44319_2025_608_MOESM11_ESM.zip › Appendix figure S1/S1B/mito.jpg]

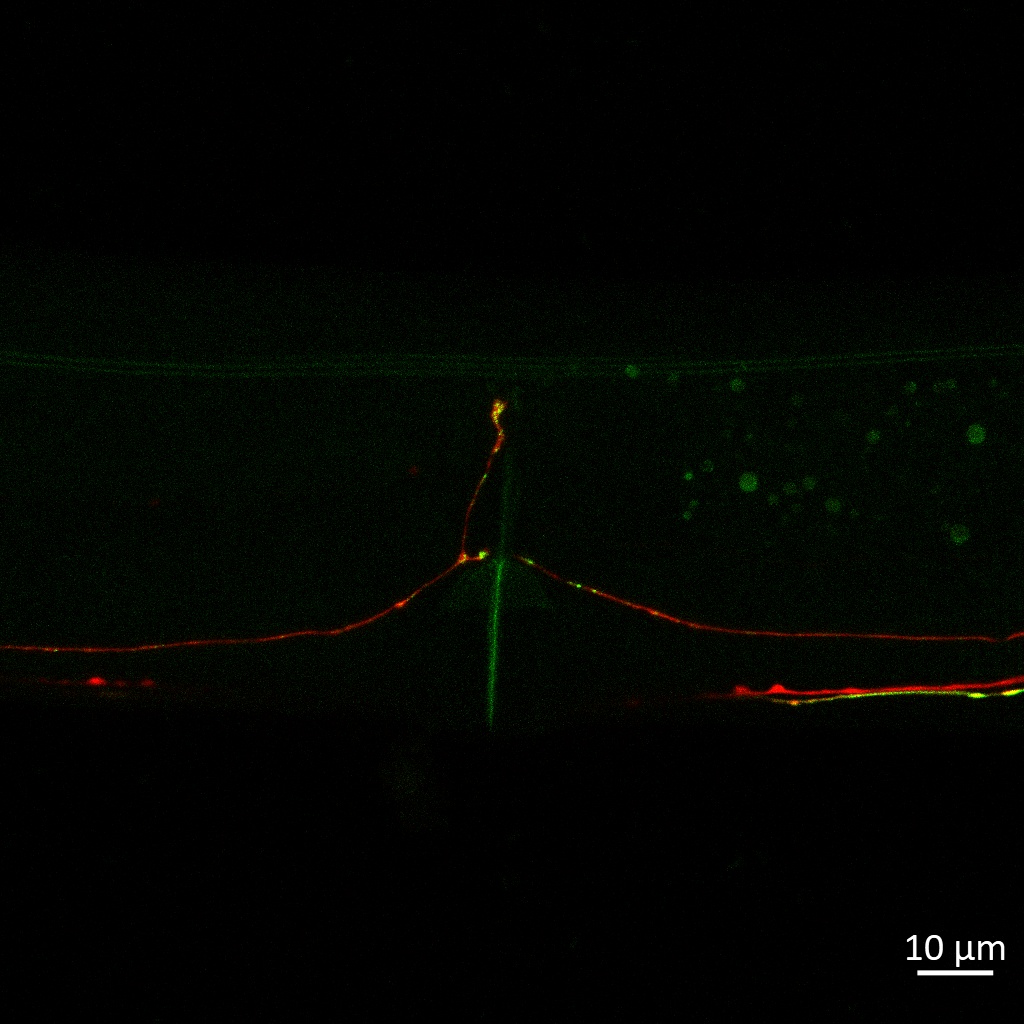

Supplement: Supplementary file 11 — Appendix and EV Figures Source Data [file 44319_2025_608_MOESM11_ESM.zip › Appendix figure S1/S1B/tgn-38.jpg]
