# Supplementary material for: Insulin and epidermal signals independently shape sexually dimorphic neurite branching in C. elegans
Source: EMBO Rep. 2025 Oct 31;26(23):5859–76. doi: 10.1038/s44319-025-00608-0 (PMC12678580; doi:10.1038/s44319-025-00608-0)
Supplement: Supplementary file 12 — Expanded View Figures [file 44319_2025_608_MOESM12_ESM.pdf]

Expanded View Figures

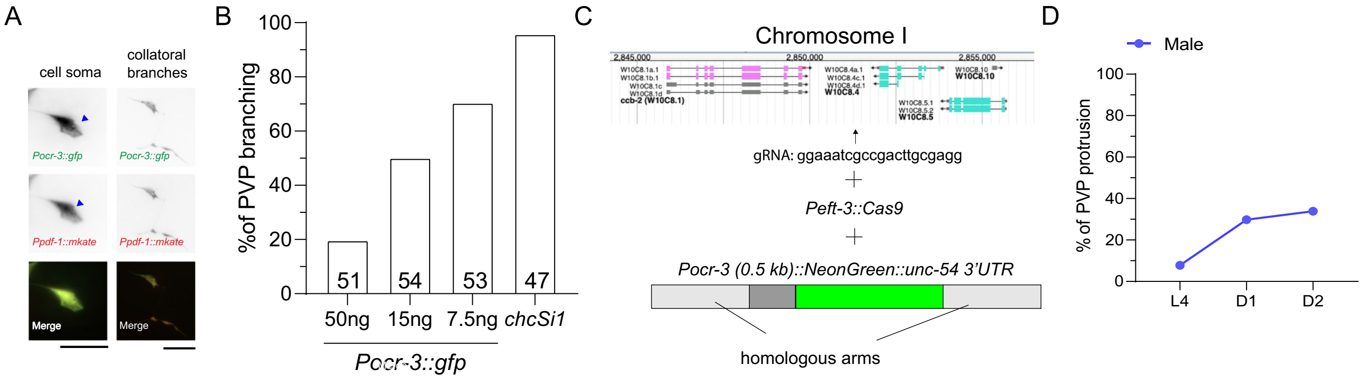

Figure EV1. Characterization of transgenic strains with PVP-specific fluorescent reporters.

(**A**) Z-projection of confocal fluorescent images of PVP neurons and markers for *pdf-1* neuropeptide in the *chcEx248[Ppdf-1::mKate; Pocr-3::gfp]*. Arrows indicate PVP branches. Scale bar = 10 µm. (**B**) Quantification of PVP branching with indicated transgenes. Fisher's exact test. *N* number and *P* value are indicated. (**C**) A schematic diagram for CRISPR-engineered single copy insertion of *Pocr-3::NeonGreen* on chromosome I. (**D**) Quantification of sexually dimorphic PVP branching at the indicated developmental stages. *N* numbers are indicated. *N* indicates the number of biological repeats in this figure. Source data are available online for this figure.

 © The Author(s)

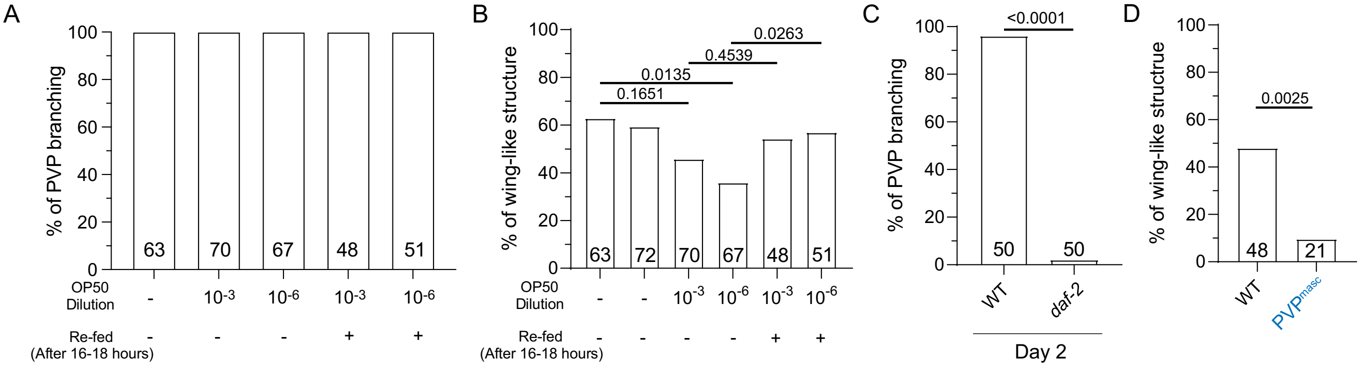

Figure EV2. Nutritional status influences PVP branch morphology.

(**A**) Quantification of wing-like branches at different nutritional statuses. (**B**) Quantification of PVP branching at different nutritional statuses. Fisher's exact test. *N* number and *P* value are indicated. (**C**) Quantification of PVP branching of D2 adults in indicated genotypes. Fisher's exact test. *N* number and *P* values are indicated. (**D**) Quantification of wing-like PVP branching in indicated genotypes. Fisher's exact test. *N* number and *P* values are indicated. *N* indicates the number of biological repeats in this figure. Source data are available online for this figure.

© The Author(s)

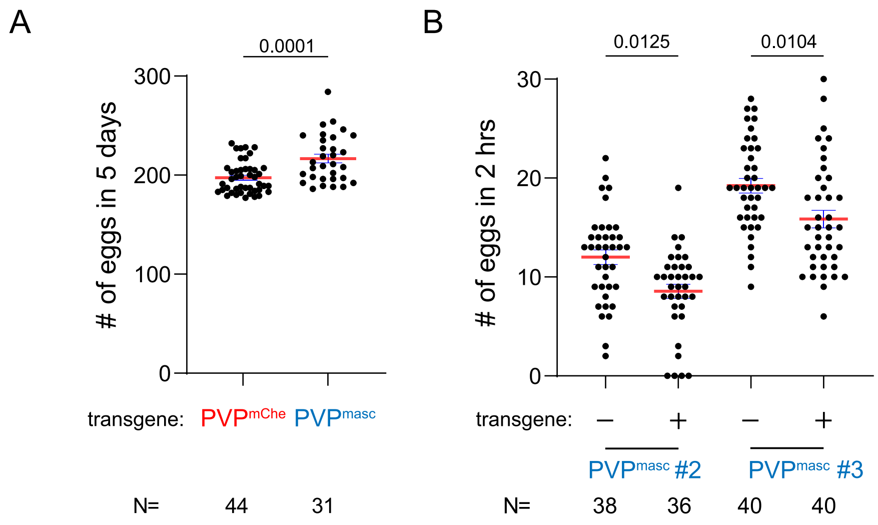

Figure EV3. Egg-laying behaviors are modulated by PVP branches.

(**A**) Quantification of total fertilized eggs with indicated genotypes. Dot represents one worm. Error bar indicates SEM. Mann–Whitney *U*-test. *N* number and *P* values are indicated. (**B**) Number of eggs laid with masculinized PVP by overexpressing FEM-3 carrying *chcEx295[Pocr-3::fem-3::SL2::mkate(7.5 ng); Pelt-2::NLS::tagBFP(50 ng)#1]* and *chcEx296[Pocr-3::fem-3::SL2::mkate(7.5 ng); Pelt-2::NLS::tagBFP(50 ng)#2]*. Error bar indicates SEM. *N* number and *P* values are indicated. One-way ANOVA with Tukey correction. Biological replicates are present in this figure. *N* indicates the number of biological repeats in this figure. Source data are available online for this figure.

 © The Author(s)
